# Prevalence of Stress in Healthcare Professionals during the COVID-19 Pandemic in Northeast Mexico: A Remote, Fast Survey Evaluation, Using an Adapted COVID-19 Stress Scales

**DOI:** 10.3390/ijerph17207624

**Published:** 2020-10-19

**Authors:** Juan Luis Delgado-Gallegos, Rene de Jesús Montemayor-Garza, Gerardo R. Padilla-Rivas, Héctor Franco-Villareal, Jose Francisco Islas

**Affiliations:** 1 Departamento de Bioquímica y Medicina Molecular, Facultad de Medicina, Universidad Autónoma de Nuevo León, Avenida Dr. Eduardo Aguirre Pequeño, Col. Mitras Centro, Monterrey, NL 64460, Mexico; jdelgado.me0174@uanl.edu.mx (J.L.D.-G.); gerardo.padillarv@uanl.edu.mx (G.R.P.-R.); 2 Instituto de Salud para el Bienestar, Clínica Psiquiátrica Dr. Everardo Neumann Peña, Carr Matehuala 8, Fracción los Olivos, Soledad de Graciano Sánchez, SLP 78430, Mexico; montemayor92@hotmail.com; 3 Althian Clinical Research, Calle Capitán Aguilar Sur 669, Col. Obispado Monterrey, NL 64000, Mexico; dr.hectorfranco@gmail.com

**Keywords:** COVID-19 Mexico, stress in healthcare professionals, COVID-19 stress scales

## Abstract

The world is currently subjected to the worst health crisis documented in modern history: an epidemic led by the novel coronavirus disease 2019 (COVID-19). At the epicenter of this crisis, healthcare professionals continue working to safeguard our well-being. To the regular high levels of stress, COVID-19 adds even more so to healthcare professionals in particular, depending on their area, specialty, and type of work. Here we investigated what are the tendencies or areas most affected. Through an adaptation of the original COVID-stress scales, we developed a remote, fast test designed for healthcare professionals in the northeastern part of Mexico, an important part of the country with economic and cultural ties to the United States. Our results showed four key correlations as highly dependent: work area–xenophobia (*p* < 0.045), work with COVID patients–traumatic stress (*p* < 0.001), total number of COVID patients per day–traumatic stress (*p* < 0.027), and total number of COVID patients–compulsive checking and reassurance. Overall, we concluded that normal levels of stress have increased (mild–moderate). Additionally, we determine that the fear of being an asymptomatic patient (potential to spread without knowing) continues being a concern.

## 1. Introduction

The emerging novel coronavirus SARS-Cov-2, which leads to the coronavirus disease 2019 (COVID-19), has affected the world profoundly. It has produced a fresh perspective on the strong and weak points hailing from every public healthcare system in the world [1,2,3,4,5,6]. COVID-19 stretches the resources of all healthcare systems to their utmost capacity, which is a grave situation, particularly since COVID-19 is now a full fledge pandemic [7]. Adding extra layers of complexity to the already difficult situation, in third-world countries there are also economic, political, and logistical situations to consider, including the availability of certain pharmaceuticals, medical equipment, and adequately trained professionals [8,9]. The overall stress of the situation is especially increased for those who attend patients, as they are not only combating the enemy at home and from the front lines, but they have to deal with the shortcomings of an overloaded system [10,11], limiting their recovery opportunity. This puts them at high risk of adverse mental health impacts, such as fatigue, anxiety, depression, and stress [12].

For all its continuous development, economic growth, and closeness to the United States, Mexico continues to be a developing democracy, with high economic disparity characterized by most of its population having to strive on low wages [13]. Interestingly, at the forefront of many past governments, health care has been a priority in Mexico, hence the development of excellent community-outreach programs, which have performed well even when underfunded [13]. This was especially true during the 2009 AH1N1 pandemic, when Mexico established a national influenza preparedness plan. This involved the development of preparedness plans for hospitals and primary care centers, strategic stockpiling, strengthening of epidemiological and laboratory surveillance, and supporting research in this area [13]. Yet the two greatest downfalls to the system have been the deficit of healthcare professionals and the hospital capacity, which given today’s circumstances has the greatest weight with regard to trying to deal at a clinical level with the pandemic [14]. Since the outbreak of COVID-19, Mexico’s health ministry began adopting a proactive position to reinforce the health system, including the national implementation of the “healthy distance”, or a 2 m distancing, between people who had to perform activities that involve coming in contact with others; a temporary suspension of non-essential activities; and the recount of daily infected patients. Thus, they are making enormous efforts in communicating preventive measures, albeit with less than favorable results [15,16]. Unfortunately, Mexico continues to be the #1 country in Latin-America in infection-to-death rate [1,17,18]. According to the official account of COVID-19 patients in Mexico, as of 31 July 2020, there were just over 424,467 total positive cases, with 46,688 deceased (>10%) [19]. The overall map of the situation, as seen here, poses important challenges to both population and healthcare providers, with an added stress to their overall mental health [5,6,20]. Even before COVID-19, according to Medina-Mora et al., the overall population in Mexico was already subjected to mental health problems like anxiety, substance abuse, and affective disorders, for which only 1 out of 10 people have received medical attention [21]. Here we will illustrate the psychological burden to healthcare providers working in a stressed-out healthcare system while under the pandemic conditions of COVID-19, which by many has been described as the worst public health crisis in generations [3,7,8].

### Psychological Impact on Healthcare Workers

Amid the COVID-19 pandemic, stress and tension have arisen amongst physicians, residents, healthcare workers, nursing staff, and related students. There is a sense of unpreparedness, coupled with a lack of vital resources and the excess of workload. All these factors contribute to physical and mental breakdown, which acts as catalyst for mental health distress [22], directly affecting attention, understanding, and the decision-making process and leaving long-lasting effects on workers’ overall wellbeing [23].

The most underestimated problem during this pandemic is the overload of work the frontline healthcare professionals undergo daily. The continuous use of personal protection equipment (PPE) in shifts of 12 to 24 h makes it difficult for the healthcare professional to stay focused on work and be capable of making optimal decisions. The physical exhaustion, emotional fatigue, and fear of self-infection or infecting someone in their family causes even more anxiety and stress. This overload of physical, mental, and emotional stress can be so significant that it can be a trigger for developing mild to severe psychiatric disorders, such as depression, anxiety, a dire need for compulsive checking for reassurance seeking, traumatic stress, and even xenophobia [24,25,26].

In a recent study by Wang et al., researchers showed the psychological impact in the initial phases of the COVID-19 pandemic; showing that over half of the population studied rated the psychological impact as moderate-to-severe, and with over a third of the group reporting moderate-to-severe anxiety [27]. Elsewhere, studies have shown that stress can induce potential benefits in the need to preserve homeostasis, as well as levels of self-motivation and survival. Counter to this, stress can induce negative emotions and effects, such as alterations in memory, cognition, learning, immune response, sleep, cardiovascular health, gastrointestinal complications, and the endocrine system [28]. Therefore, understanding the balance is key to maintaining a healthy environment and the adequate mental state of the person. This is especially true for frontline healthcare workers, as studies have shown that workers directly engaged with the diagnosis, treatment, and care of patients with COVID-19 associate with a higher risk of symptoms of depression [29]. This study proposes to help fill the void of understanding how trained health professional with different specialties and working in different areas cope with the stress or have adapted to the current work environment [21]. We have adapted the COVID-19 stress scales [26] as a means to help identify the circumstances that most likely affect health care professionals.

## 2. Materials and Methods

This study proposes the application of the COVID-19 stress scales (CSS). The CSS was established and validated in population samples from Canada and the United States. These scales were adapted for the Spanish-speaking healthcare professional community in Mexico, based on the 36-item questionnaire CSS developed by Taylor et al., which is used to assess stress and anxiety symptoms in daily life [26]. Our questionnaire analyzes six psychometric areas of the CSS: danger and contamination fears (evaluated together as area 1), fears about economic consequences (area 2), xenophobia (area 3), compulsive checking and reassurance seeking (area 4), and traumatic stress symptoms (area 5), all related to COVID-19. The adapted questionnaire is shown on Table 1.

The questionnaire was written using MS Forms (Microsoft Corporation, Redwood, WA, United States), and was applied remotely through a web link. The test was distributed to healthcare professionals hailing from the northeast part of Mexico, mainly from Monterrey, San Luis Potosi, and the Mexico–United States border towns of Nuevo Laredo and Matamoros, through electronic means, such as email invitations and local medical social media groups. The questionnaire was applied during a 6-week period spanning from July to August 2020, the period with the highest peak of daily cases reported, according to the Mexico health ministry [30,31]. All subjects acknowledged being >18 years old and gave their consent for inclusion before participating in the study. A Likert scale format was used with increasing point values [32]. All statistical analysis correlations were calculated using IBM SPSS Statistics for Windows, version 23.0 (IBM Corp., Armonk, NY, USA) with Pearson’s chi-squared and an R (R Core Team, Vienna, Austria) ratio of 0.05 to evaluate the prevalence of the alteration of mental health in health professionals attending COVID-19 patients in Mexico.

Briefly, we calculated the frequency of answers in relation to categories, areas, and other variables. We then correlated the answers to the number of points in each section. The resulting ranges were classified in the following categories: 0–5 = absent, 6–11 = mild, 12–17 = moderate, and 18–24 = severe. Next, we made a general scale to assess COVID-19 stress, using cumulative scores for each section: 0–35 = absent, 36–71 = mild, 72–107 = moderate, and 108–144 = severe. An extra question was added to the first section of the questionnaire, measuring “the fear of being an asymptomatic patient” (FOBAP), which was scored independently so as not to alter the structure of the original CSS. The scores for FOBAP on a scale of 0–4 and a classification was correlated to the number of points scored in the question: 0 = absent, 1 = normal, 2 = mild, 3 = moderate, and 4 = severe. Other items regarding the healthcare professionals, corresponding to the level or type of training, specialties, areas of work, number of COVID-19 patients attended per day, and if the subjects had themselves a previous diagnose of COVID-19, were added, as well as their willingness to continue participating in follow-up questionnaires, totaling 45 items.

The study was conducted in accordance with the Declaration of Helsinki, and the protocol was approved by the Ethics Committee of Hospital La Misión, Monterrey NL. México. Protocol #PSY-CSS-ESP-001.

## 3. Results

From 110 participants recruited remotely, six presented exclusion factors, i.e., declining to take part in the test. Participants were not required to answer all sections to advance through the questionnaire. Out of the total participants that answered the CSS, we obtained the following results.

**Figure 1:**
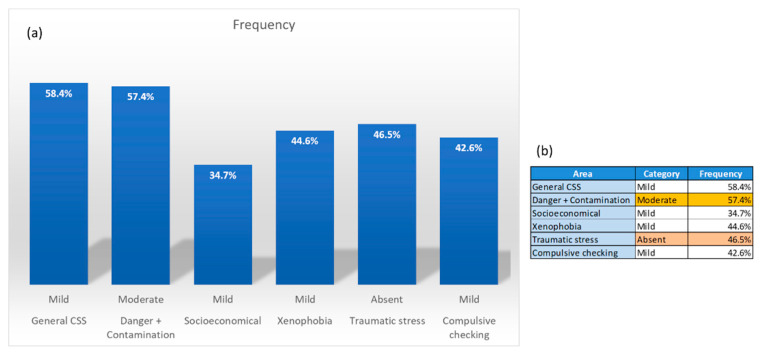
Most representative COVID-19 stress level frequency for healthcare providers by area. (**a**) Graph of frequencies in relation to areas. (**b**) Table showing the most representative frequencies in each area. Areas of socioeconomics, xenophobia, and compulsive checking show levels of mild stress, Danger and contamination show levels of moderate stress, and traumatic stress shows an absence of stress.

First, from the evaluated stress-level frequency for all healthcare professionals, in accordance to the general areas, the percentage of frequency was as follows: in area 1, 43% scored “absent”, 22.8% “mild”, 57.4% “moderate”, and 16.8% “severe”; in area 2, 29.7% scored “absent”, 34.7% “mild”, 23.8% “moderate”, and 11.9% “severe”; in area 3, "14.9% scored “absent”, 44.6% “mild”, 28.7% “moderate”, and 11.9% “severe”; in area 5, 46.5% scored “absent”, 24.8% “mild”, 20.8% “moderate”, and 7.9% “severe”; in the compulsive checking and reassurance seeking area, area 6, 25.7% scored “absent”, 42.6% “mild”, 21.8% “moderate”, and 9.9% “severe” (Table 2). The most representative values are presented in Figure 1.

Next, we analyzed the data for correlations, separating it into different categories. First, we analyzed by profession: physician resident, physician, medical student, physician in community service (a medical student who has finished the required medical school training in Mexico and is doing a compulsory one-year internship at a local community hospital or health facility, after which time the student is awarded their medical license), nurse, and others. Next, we analyzed by work area: pediatrics, first-line healthcare provider (a common term in Spanish for certain healthcare providers in a designated area that falls into primary health provider and front-line healthcare provider, both common terms in English-speaking countries; emergency care providers fall in a separate category), COVID-19 designated area, internal medicine, intensive care unit (ICU), radiology, obstetrics and gynecology (OBGYN), surgical area, emergency room (ER), and others. Then, we analyzed for previous COVID-19 diagnosis, work with COVID-19 patients, total number of COVID-19 patients per day (separate analysis based on the number of patients), and finally, the FOBAP question.

We began by looking at the category of profession, correlating it to the total CSS (*p* < 0.977), and showed the following correlations: area 1, *p* < 0.840; area 2, *p* < 0.367; area 3, *p* < 0.931; area 4, *p* < 0.108; and area 5, *p* < 0.524 (Supplementary Table S1). Next, we separated data into medical and nursing professionals, and correlated their CSS results (*p* < 0.849); for individual areas, the results showed the following correlations: area 1, *p* < 0.629; area 2, *p* < 0.321; area 3, *p* < 0.700; area 4, *p* < 0.677; area 5, *p* < 0.357 (Supplementary Table S2). We then analyzed by work area, and correlated that to the CSS (*p* < 0.275); for individual areas, the results showed the following correlations: for area 1, *p* < 0.998; area 2, *p* < 0.489; area 3, *p* < 0.045; area 4, *p* < 0.144; and area 5, *p* < 0.2507 (Supplementary Table S3). We further analyzed the data by positive diagnosis to COVID-19, with results for the total CSS (*p* < 0.664), and for individual areas as follows: area 1, *p* < 0.542; area 2, *p* < 0.664; area 3, *p* < 0.653; area 4, *p* < 0.781; and area 5, *p* < 0.666 (Supplementary Table S4). We then analyzed the data as related to whether professionals work with COVID-19-positive patients, with a result for the total CSS of *p* < 0.303, and for individual areas as follows: area 1, *p* < 0.266; area 2, *p* < 0.786 area 3, *p* < 0.553; area 4, *p* < 0.001; and area 5, *p* < 0.121 (Supplementary Table S5). We then reanalyzed by the number of patients the subjects work with, with a result for the total CSS of *p* < 0.076. For individual areas, as follows, the results were area 1, *p* < 0.122; area 2, *p* < 0.521; area 3, *p* < 0.077; area 4, *p* < 0.027; and area 5, *p* < 0.047 (Supplemental Table S6). Finally, we analyzed the data in relation to the FOBAP question; results for the total CSS were *p* < 0.001, and the other results were as follows: area 1, *p* < 0.003; area 2, *p* < 0.638; area 3, *p* < 0.047; area 4, *p* < 0.002; and area 5, *p* < 0.024 (Supplementary Table S7). Table 3 shows Pearson’s chi-squared test for all tested correlations.

## 4. Discussion

From the data collected and looking at the frequency of response by area, our results, as seen on Table 2 and Figure 1, show that the areas of socioeconomical consequences, xenophobia, and compulsive checking scored responses potentially representing mild stress; meanwhile, danger and contamination scores tended to indicate moderate stress, and strikingly traumatic stress scored as absent. We should note that this is a first approximation that encompasses all data with no breakdown. According to this analysis, the most predominant result is mild levels of stress because of the COVID-19 pandemic. Theoretically, an absent result of COVID-19-induced stress is what one would expect under normal conditions. We must emphasize that daily lives and activities, particularly in “naturally high-stressful jobs” like health care [33,34,35], due to the height of their particular level of individual stress, should never be discarded when evaluating a person. However, our observations are on the effects of the pandemic over these normal levels of stress. Remarking that the COVID-19 pandemic has been one of the worst pandemics in recorded history [36,37,38], it is understandable that the levels of evaluated stress would rise. Comparable to our results, a nationwide study on healthcare professionals by Mohd Fauzi et al. showed high levels of anxiety, fatigue, and depression. In addition, lower levels of inter-shift recovery due to the high work demand worsened mental stress, while recovery experiences protected mental health [21]. Furthermore, in a systematic review by Luken et al., researchers pointed out that even under “normal” conditions, job burnout related to stress was particularly high for healthcare professionals. Considering the pandemic, it is not surprising that the apparent stress for most areas have risen. We should also note that these researchers mentioned that healthcare and other professionals presented with de-stressing activities, such as mindfulness seminars or other relaxing techniques, would present considerably less burnout [33,39]. As stress rises across the board, it was predictable to see that the areas of danger and contamination would have shown even higher levels of stress, which for us translated into potentially moderate levels. What we found interesting is traumatic stress, as it scored as potentially absent from a general view. One likely scenario that may be taking place is the beginning of “normalization” of conditions at work. As guidelines have improved, work conditions for professionals could experience a relief surpassing that initial traumatic stress phase [5,40]. Next, as we begin to break down and make correlations, we will see how more individualized populations analyzed for different variables statistically correlated with different areas affected by COVID stress. As we embark further into the outcome of the pandemic, it would be interesting to ask if this “normalization” can spill into the other categories, and how this varies along with the reduction in the number of cases as the global situation improves. Yet, for our initial work, this is the picture we have during the months of July–August, almost 6 months after the first reported case in northeast Mexico [41].

Different healthcare professionals perceive stress in different ways, in part because of their training (type, experience, and level), their current work area, the type and number of patients they work with every day, and other related variables. When analyzing the data, we took these variables into consideration, in order to better understand the tendencies of our results. In Table 3, we can observe four interesting statistical correlations in three areas when crossed with categories in the following manner: work area–xenophobia (*p* < 0.045, Supplementary Table S3), Work with COVID patients–traumatic stress (*p* < 0.001, Supplementary Table S5), total number of COVID patients per day–traumatic stress (*p* < 0.027, Supplementary Table S6), and total number of COVID patients–compulsive checking (*p* < 0.024, Supplementary Table S6).

Our first observation relates to the correlation of work area–xenophobia, where 41.7% of the total response showed a tendency towards a mild level of stress. Professionals working in “others” (all areas not accounted for in our work area breakdown) category, as well as in a COVID-designated area, had the highest proportion of stress, totaling a combined 53% of the response. Within COVID-designated areas, mild levels of stress represented 72.7% of the total responses. Mild levels of “others” represented 70%. Results of moderate levels of stress was the second highest category, representing 28.2% with first-line healthcare providers and 42% with internal medicine. The results for these categories continue to affirm the mild tendency of fear towards people from outside the state. Although the overall tendency is mild levels of stress, these results seem to associate with potential migration. For the case of the border towns, border crossings are normal activities, yet in past several years there have been noticeable waves of migrants from outside the country seeking asylum to the United States, coming from central and south America [42]. Even though these waves began before the current pandemic, these asylum seekers represent vulnerable groups with low or no income. Therefore, it is reasonable to think COVID-19 outbreaks within these vulnerable groups would spread fast [43,44,45]. In the cases of Monterey and San Luis Potosi, they are both major metropolitan cities geographically at close distance with the United States. Both cities have major airports and major highways, which makes it easy to have a high effluence of regional and out-of-state visitors.

Results for traumatic stress proved to be highly dependent upon two categories: work with COVID patients (Supplementary Table S5) and the number of patients worked with (Supplementary Table S6). Previously, we mentioned that the traumatic stress score was overwhelmingly absent (Table 2 and Figure 1), and when broken down by categories, this continues to hold true. Of the total of participants who took the test, 58% answered “yes” to working directly with COVID-19 patients; furthermore, the participants from the participants who work with COVID-19 (35%) scored potentially absent for levels of stress, and when compared to their counterparts (not working with COVID-19 patients) the result more than doubled, with 72.1% scoring “absent”. Remarkably, healthcare professionals who do work with COVID-19 positive patients scored higher in the “mild” category with 45%, which was almost four times higher than those who do not, who scored 11.6%. When combined the “absent” result, these scores represented 50.5% (40% those working with COVID-19 patients, and 60% not working with COVID-19 patients). Meanwhile, the “mild” result was 31.1% (84% working with COVID-19 patients, and 16% not working with COVID-19 patients). These results show that there is a high sense of relief for stress tendencies by not working directly with COVID-19 patients. Nonetheless, when it comes to those working with COVID-19 patients, the tendency is to score low in the overall stress level, but the perceivable difference is almost 10% higher for the “mild” result—a significant difference. When we analyzed by number of patients, slightly more than half (50.8%) scored “absent” (albeit 71% represented no patients), while the “mild” category only scored 31.1%. We also found that 50% of professionals working with more than 20 COVID-19 patients scored “absent” and 41.7% scored “mild”, totaling 91.7% of that group. Although this was almost an outlying situation, other professionals working with COVID-19 had similar tendencies, but in all other cases, the “mild” result had the highest frequency, typically followed by “absent”. Only the group of 10 to 20 patients (lowest number of professionals) per day showed a higher tendency in the moderate results. Although more analysis is needed, the 10 to 20 patients per day ratio brings up some interesting questions, such as, is this the range where professionals work with more critical patients? Is the time spent with these patients sufficient to induce moderate stress? What other conditions play into this tendency? Along with stress, the compulsive need for checking and reassurance (Supplementary Table S6) also depended on the number of patients seen per day, and as expected, there were similar tendencies. One interesting observation found when looking at the group with a low number of patients (1 to 5) is that they scored 30.3% in the “moderate” level and 39.8% in the “mild” level, rivaled only by the 10 to 20 patients per day, which was 50% in the “moderate” and 33.3% in the “mild” category. Therefore, the need to check and reassure oneself potentially depends on the number of patients. As we stated before, this is rational, as the number of patients also seems to induce a sense of traumatic stress. Taylor et al. describes that in time of pandemic, people exhibit anxiety-related responses—both traumatic stress and the need for checking and reassurance fall into these behaviors, as the second is a self-defense mechanism to mitigate the feelings developing during stressful situations [26].

## 5. Conclusions

We have shown here the application of an adapted version of the CSS for healthcare professionals in the northeast of Mexico. Healthcare professionals attending COVID-19 showed mostly mild and, in particular cases, moderate stress in different areas, with traumatic stress, xenophobia, and compulsive checking being the most predominant in their daily lives. Alongside this, there is a fear of being an asymptomatic patient, as this condition might mean they themselves are a source of infection to the community and to their patients. Unfortunately, our study is limited to a small number of participants from different work areas and specialties. Understandably, some professionals with a higher level of stress might be unwilling to participate, due to their high demand of work, while minimizing their own malaise when they perceive that the malaise in the patients is higher. Although the variable of gender was not used for analysis, as this was a small-scale study, 57% of the participants were male and 43% were female. A larger number of participants per area and more specialties are needed to be thoroughly conclusive. However, our results show important tendencies that should be addressed with regard to how different areas in the medical field are being affected. In addition, levels of stress and potential burnout should be an essential focus at an administrative level, inorder to maintain a healthy team of healthcare professionals.

## Figures and Tables

**Table 1:** Structure and adaptation of the COVID-19 stress scales (CSS) [26] for Spanish-speaking healthcare providers.

Initial Questions	
1	¿Desea usted participar en el cuestionario?
2	¿Cuál es su profesión?
3	¿En qué área trabaja?
4	¿Trabaja usted con pacientes con coronavirus?
5	¿Con cuántos pacientes trabaja diariamente?
Section 1 (Danger) **	
6	Estoy preocupado por contraer el virus
7	Estoy preocupado de ya tener el virus y ser asintomático *
8	Me preocupa que la higiene básica (por ejemplo, el lavado de manos) no sea suficiente para mantenerme a salvo del virus
9	Me preocupa que nuestro sistema de salud no pueda mantenerme a salvo del virus
10	Me preocupa no poder mantener a mi familia a salvo del virus
11	Me preocupa que nuestro sistema de salud no pueda proteger a mis seres queridos
12	Me preocupa que el distanciamiento social no sea suficiente para mantenerme a salvo del virus
Section 2 (Socialeconomical)	
13	Me preocupa que las tiendas de comestibles se queden sin comida
14	Me preocupa que las tiendas de comestibles se queden sin remedios para el resfriado o la gripe
15	Me preocupa que las farmacias se queden sin medicamentos recetados
16	Me preocupa que las tiendas de comestibles se queden sin agua
17	Me preocupa que las tiendas de comestibles se queden sin productos de limpieza o desinfectantes.
18	Me preocupa que las tiendas de comestibles cierren
Section 3 (Xenophobia)	
19	Me preocupa que personas fuera del estado estén propagando el virus.
20	Me preocupa que las personas que conozco, que viven fuera de mi estado, puedan tener el virus.
21	Me preocupa entrar en contacto con personas fuera del estado porque pueden tener el virus.
22	Me preocupa que personas extranjeras estén propagando el virus porque no están tan limpios como nosotros
23	Si fuera a un restaurante especializado en alimentos extranjeros, me preocuparía contraer el virus
24	Si estuviera en un elevador con un grupo de extranjeros, me preocuparía que estén infectados con el virus.
Section 4 (Fear of Contamination) **	
25	Me preocupa que las personas a mi alrededor me infecten con el virus
26	Me preocupa que si tocara algo en un espacio público (por ejemplo, pasamanos, manija de la puerta), pueda contraer el virus
27	Me preocupa que, si alguien tosiera o estornudara cerca de mí, podría contraer el virus.
28	Me preocupa que pueda contraer el virus al manejar dinero o usar una máquina de tarjeta de débito/crédito
29	Estoy preocupado por hacer transacciones en efectivo
30	Me preocupa que mi paquetería/correo haya sido contaminado por los manejadores de correo
Section 5 (Traumatic stress)	
31	Tuve problemas para dormir porque me preocupaba el virus
32	Tuve malos sueños sobre el virus
33	Pensé en el virus cuando no quise
34	Aparecieron en mi mente, contra mi voluntad, imágenes mentales inquietantes sobre el virus
35	Tuve problemas para concentrarme porque seguía pensando en el virus
36	Los recordatorios del virus me provocaron reacciones físicas, como sudoración o latidos fuertes del corazón.
Section 6 (Compulsive Checking)	
37	Reviso ubicaciones en redes sociales sobre COVID-19
38	Reviso videos de YouTube sobre COVID-19
39	Solicitó tranquilidad a amigos o familiares sobre COVID-19
40	Reviso mi propio cuerpo en busca de signos de infección (p. Ej., Tomando mi temperatura)
41	Pido consejo a los profesionales de la salud (por ejemplo, médicos o farmacéuticos) sobre COVID-19
42	Busco en Internet tratamientos para COVID-19
Final questions for future follow-up	
43	Ha sido diagnosticado con COVID 19
44	¿Le interesaría en un futuro participar en un cuestionario para seguimiento de su salud mental? ***
45	Le agradecemos su interés y le pedimos, por favor nos deje una dirección de correo electrónico ***

* Fear of being an asymptomatic patient (FOBAP). ** Danger and Fear of Contamination were evaluated together as area 1. *** Follow-up interest.

**Table 2:** COVID-related stress frequency for healthcare providers correlated to the general CSS and studied individual areas.

	CSS General Score				COVID Danger + Contamination			
	*N* Observed	*N* Expected	Residue	%	*N* Observed	*N* Expected	Residue	%
Absent	9	25.3	−16.3	8.9	3	25.3	−22.3	3.0
Mild	59	25.3	33.8	58.4	23	25.3	−2.3	22.8
Moderate	28	25.3	2.8	27.7	58	25.3	32.8	57.4
Severe	5	25.3	−20.3	5.0	17	25.3	−8.3	16.8
	**COVID Socioeconomic Consequences**				**COVID Xenophobia**			
	***N* Observed**	***N* Expected**	**Residue**	**%**	***N* Observed**	***N* Expected**	**Residue**	**%**
Absent	30	25.3	4.8	29.7	15	25.3	−10.3	14.9
Mild	35	25.3	9.8	34.7	45	25.3	19.8	44.6
Moderate	24	25.3	−1.3	23.8	29	25.3	3.8	28.7
Severe	12	25.3	−13.3	11.9	12	25.3	−13.3	11.9
	**COVID Traumatic Stress**				**COVID Compulsive Checking**			
	***N* Observed**	***N* Expected**	**Residue**	**%**	***N* Observed**	***N* Expected**	**Residue**	**%**
Absent	47	25.3	21.8	46.5	26	25.3	0.8	25.7
Mild	25	25.3	−0.3	24.8	43	25.3	17.8	42.6
Moderate	21	25.3	−4.3	20.8	22	25.3	−3.3	21.8
Severe	8	25.3	−17.3	7.9	10	25.3	−15.3	9.9
		CSS	area 1 + 4	area 2	area 3	area 5	area 6	
Pearson’s chi-square		72.109 ^a^	64.980 ^a^	11.673 ^a^	27.119 ^a^	31.238 ^a^	22.129 ^a^	
gl		3	3	3	3	3	3	
Asymptotic sig.		<0.001	<0.001	0.009	<0.001	<0.001	<0.001	

^a^ 0 cells (0.0%) have an expected frequency lower than 5. The expected minimum frequency is 25.3.

**Table 3:** Overview of all resulting Pearson´s chi-square for all tested correlations.

	Total CSS	Danger + Contamination	Socioeconomic Consequences
Medical professions	*p <* 0.977	*p <* 0.840	*p <* 0.367
Medical vs. nursing professionals	*p <* 0.849	*p <* 0.629	*p <* 0.321
Work area	*p <* 0.275	*p <* 0.998	*p <* 0.498
Previous COVID-positive diagnosis	*p <* 0.664	*p <* 0.542	*p <* 0.664
Work with COVID patients	*p <* 0.303	*p <* 0.266	*p <* 0.786
Total number of COVID patients per day	*p <* 0.076	*p <* 0.122	*p <* 0.521
Fear of being an asymptomatic patient (FOBAP)	*p <* 0.001	*p <* 0.003	*p <* 0.638
	**Xenophobia**	**Traumatic Stress**	**Compulsive Checking**
Medical professions	*p <* 0.931	*p <* 0.108	*p <* 0.524
Medical vs. nursing professionals	*p <* 0.700	*p <* 0.677	*p <* 0.357
Work area	*p <* 0.045	*p <* 0.144	*p <* 0.250
Previous COVID-positive diagnostic	*p <* 0.653	*p <* 0.781	*p <* 0.666
Work with COVID patients	*p <* 0.553	*p <* 0.001	*p <* 0.121
Total number of COVID patients per day	*p <* 0.077	*p <* 0.027	*p <* 0.047
Fear of being an asymptomatic patient (FOBAP)	*p <* 0.047	*p <* 0.002	*p <* 0.024

## Supplementary Materials

The following are available online at https://www.mdpi.com/1660-4601/17/20/7624/s1, Table S1: COVID stress scales test correlations by training. Table S2: COVID stress scales test correlations for medical/nursing. Table S3: COVID Stress Scales test correlations by work area. Table S4: COVID stress scales test correlations by COVID diagnosis. Table S5: COVID stress scales test correlations by work with (or without) COVID+ patients. Table S6: COVID stress scales test correlations by work, including number of COVID+ patients. Table S7: FOBAP correlations by CSS areas. Table S8: Development of the scale construction scores for FOBAP analysis.
