# Supplementary material for: Prevalence of Stress in Healthcare Professionals during the COVID-19 Pandemic in Northeast Mexico: A Remote, Fast Survey Evaluation, Using an Adapted COVID-19 Stress Scales
Source: Int J Environ Res Public Health. 2020 Oct 19;17(20):7624. doi: 10.3390/ijerph17207624 (PMC7593933; doi:10.3390/ijerph17207624)
Supplement: Supplementary file 1 [file ijerph-17-07624-s001.zip › supp table/supp table 1.docx]

| **Profession CSS** |  |  | | | |  |  | | **Profession Danger + Contamination** | | |  | | | |  |  |
| --- | --- | --- | --- | --- | --- | --- | --- | --- | --- | --- | --- | --- | --- | --- | --- | --- | --- |
| Training |  | ABSENT | MILD | MODERATE | SEVERE | Total |  | | Training | |  | ABSENT | MILD | MODERATE | SEVERE | Total |  |
| Physician Resident | Frequency | 2 | 10 | 3 | 0 | 15 |  | | Physician Resident | | Frequency | 2 | 2 | 10 | 1 | 15 |  |
|  | Percentage (%) | 13.3% | 66.7% | 20.0% | 0.0% | 100.0% |  | |  | | Percentage (%) | 13.3% | 13.3% | 66.7% | 6.7% | 100.0% |  |
| Physician | Frequency | 6 | 40 | 18 | 5 | 69 |  | | Physician | | Frequency | 5 | 20 | 32 | 12 | 69 |  |
|  | Percentage (%) | 8.7% | 58.0% | 26.1% | 7.2% | 100.0% |  | |  | | Percentage (%) | 7.2% | 29.0% | 46.4% | 17.4% | 100.0% |  |
| Medical Student | Frequency | 0 | 1 | 1 | 0 | 2 |  | | Medical Student | | Frequency | 0 | 0 | 2 | 0 | 2 |  |
|  | Percentage (%) | 0.0% | 50.0% | 50.0% | 0.0% | 100.0% |  | |  | | Percentage (%) | 0.0% | 0.0% | 100.0% | 0.0% | 100.0% |  |
| Physician in community service | Frequency | 0 | 2 | 2 | 0 | 4 |  | | Physician in community service | | Frequency | 0 | 1 | 3 | 0 | 4 |  |
|  | Percentage (%) | 0.0% | 50.0% | 50.0% | 0.0% | 100.0% |  | |  | | Percentage (%) | 0.0% | 25.0% | 75.0% | 0.0% | 100.0% |  |
| Nursing | Frequency | 1 | 5 | 4 | 0 | 10 |  | | Nursing | | Frequency | 0 | 2 | 7 | 1 | 10 |  |
|  | Percentage (%) | 10.0% | 50.0% | 40.0% | 0.0% | 100.0% |  | |  | | Percentage (%) | 0.0% | 20.0% | 70.0% | 10.0% | 100.0% |  |
| Other | Frequency | 0 | 1 | 1 | 0 | 2 |  | | Other | | Frequency | 0 | 1 | 1 | 0 | 2 |  |
|  | Percentage (%) | 0.0% | 50.0% | 50.0% | 0.0% | 100.0% |  | |  | | Percentage (%) | 0.0% | 50.0% | 50.0% | 0.0% | 100.0% |  |
| Total | Frequency | 9 | 59 | 29 | 5 | 102 |  | | Total | | Frequency | 7 | 26 | 55 | 14 | 102 |  |
|  | Percentage (%) | 8.8% | 57.8% | 28.4% | 4.9% | 100.0% |  | |  | | Percentage (%) | 6.9% | 25.5% | 53.9% | 13.7% | 100.0% |  |
|  | Value | df | Sig. Asymptotic (bilateral) | | |  |  | |  | | Value | df | Sig. Asymptotic (bilateral) | | |  |  |
| Pearson Chi-square | 6.142^a^ | 15 | 0.977 |  |  |  |  | | Pearson Chi-square | | 9.667^a^ | 15 | 0.840 |  |  |  |  |
| Verisimilitude | 8.067 | 15 | 0.921 |  |  |  |  | | Verisimilitude | | 12.240 | 15 | 0.661 |  |  |  |  |
| linear association | 0.735 | 1 | 0.391 |  |  |  |  | | linear association | | 0.571 | 1 | 0.450 |  |  |  |  |
| N cases | 102 |  |  |  |  |  |  | | N cases | | 102 |  |  |  |  |  |  |
| a. 19 cells (79.2%) have an expected frequency lower than 5.The expected minimum frequency is10. | | | | | | | |  | | a. 19 cells (79.2%) have an expected frequency lower than 5. The expected minimum frequency is14. | | | | | | | |

|  |  |  |  |  |  |  |  |  |  |  |  |  |  |  |
| --- | --- | --- | --- | --- | --- | --- | --- | --- | --- | --- | --- | --- | --- | --- |
| **Profession Socioeconomical** |  |  | | | |  |  | **Profession Xenophobia** |  |  | | | | Total |
| Training |  | ABSENT | MILD | MODERATE | SEVERE | Total |  | Training |  | ABSENT | MILD | MODERATE | SEVERE |  |
| Physician Resident | Frequency | 7 | 3 | 3 | 2 | 15 |  | Physician Resident | Frequency | 3 | 8 | 2 | 2 | 15 |
|  | Percentage (%) | 46.7% | 20.0% | 20.0% | 13.3% | 100.0% |  |  | Percentage (%) | 20.0% | 53.3% | 13.3% | 13.3% | 100.0% |
| Physician | Frequency | 27 | 25 | 13 | 4 | 69 |  | Physician | Frequency | 13 | 28 | 21 | 7 | 69 |
|  | Percentage (%) | 39.1% | 36.2% | 18.8% | 5.8% | 100.0% |  |  | Percentage (%) | 18.8% | 40.6% | 30.4% | 10.1% | 100.0% |
| Medical Student | Frequency | 0 | 1 | 1 | 0 | 2 |  | Medical Student | Frequency | 0 | 1 | 1 | 0 | 2 |
|  | Percentage (%) | 0.0% | 50.0% | 50.0% | 0.0% | 100.0% |  |  | Percentage (%) | 0.0% | 50.0% | 50.0% | 0.0% | 100.0% |
| Physician in community service | Frequency | 1 | 3 | 0 | 0 | 4 |  | Physician in community service | Frequency | 1 | 1 | 2 | 0 | 4 |
|  | Percentage (%) | 25.0% | 75.0% | 0.0% | 0.0% | 100.0% |  |  | Percentage (%) | 25.0% | 25.0% | 50.0% | 0.0% | 100.0% |
| Nursing | Frequency | 2 | 4 | 3 | 1 | 10 |  | Nursing | Frequency | 4 | 3 | 2 | 1 | 10 |
|  | Percentage (%) | 20.0% | 40.0% | 30.0% | 10.0% | 100.0% |  |  | Percentage (%) | 40.0% | 30.0% | 20.0% | 10.0% | 100.0% |
| Other | Frequency | 0 | 0 | 2 | 0 | 2 |  | Other | Frequency | 0 | 1 | 1 | 0 | 2 |
|  | Percentage (%) | 0.0% | 0.0% | 100.0% | 0.0% | 100.0% |  |  | Percentage (%) | 0.0% | 50.0% | 50.0% | 0.0% | 100.0% |
| Total | Frequency | 37 | 36 | 22 | 7 | 102 |  | Total | Frequency | 21 | 42 | 29 | 10 | 102 |
|  | Percentage (%) | 36.3% | 35.3% | 21.6% | 6.9% | 100.0% |  |  | Percentage (%) | 20.6% | 41.2% | 28.4% | 9.8% | 100.0% |
|  | Value | df | Sig. Asymptotic (bilateral) | | |  |  |  | Value | df | Sig. Asymptotic (bilateral) | | |  |
| Pearson Chi-square | 16.235^a^ | 15 | 0.367 |  |  |  |  | Pearson Chi-square | 7.817^a^ | 15 | 0.931 |  |  |  |
| Verisimilitude | 16.505 | 15 | 0.349 |  |  |  |  | Verisimilitude | 9.110 | 15 | 0.872 |  |  |  |
| linear association | 2.836 | 1 | 0.092 |  |  |  |  | linear association | 0.394 | 1 | 0.530 |  |  |  |
| N cases | 102 |  |  |  |  |  |  | N cases | 102 |  |  |  |  |  |
| a. 19 cells (79.2%) have an expected frequency lower than 5. The expected minimum frequency is14. |  |  |  |  |  |  |  | a. 19 cells (79.2%) have an expected frequency lower than 5. The expected minimum frequency is20. |  |  |  |  |  |  |

|  | | | |  | |  | |  | |  | |  | |  | |  |  |  |  |  |  |  |
| --- | --- | --- | --- | --- | --- | --- | --- | --- | --- | --- | --- | --- | --- | --- | --- | --- | --- | --- | --- | --- | --- | --- |
| **Profession Traumatic stress** |  |  | | | | | | | Total | |  | | **Profession Compulsive** | |  | | CLASIFCOMPULSIV | | | |  |  |
| Training |  | ABSENT | MILD | | MODERATE | | SEVERE | |  |  |  | | Training | |  | | .00 | 2.00 | 3.00 | 4.00 | Total |  |
| Physician Resident | Frequency | 12 | 2 | | 1 | | 0 | | 15 | |  | | Physician Resident | | Frequency | | 8 | 5 | 2 | 0 | 15 |  |
|  | Percentage (%) | 80.0% | 13.3% | | 6.7% | | 0.0% | | 100.0% | |  | |  | | Percentage (%) | | 53.3% | 33.3% | 13.3% | 0.0% | 100.0% |  |
| Physician | Frequency | 34 | 20 | | 8 | | 7 | | 69 | |  | | Physician | | Frequency | | 19 | 30 | 15 | 5 | 69 |  |
|  | Percentage (%) | 49.3% | 29.0% | | 11.6% | | 10.1% | | 100.0% | |  | |  | | Percentage (%) | | 27.5% | 43.5% | 21.7% | 7.2% | 100.0% |  |
| Medical Student | Frequency | 1 | 0 | | 1 | | 0 | | 2 | |  | | Medical Student | | Frequency | | 1 | 0 | 1 | 0 | 2 |  |
|  | Percentage (%) | 50.0% | 0.0% | | 50.0% | | 0.0% | | 100.0% | |  | |  | | Percentage (%) | | 50.0% | 0.0% | 50.0% | 0.0% | 100.0% |  |
| Physician in community service | Frequency | 0 | 4 | | 0 | | 0 | | 4 | |  | | Physician in community service | | Frequency | | 1 | 2 | 0 | 1 | 4 |  |
|  | Percentage (%) | 0.0% | 100.0% | | 0.0% | | 0.0% | | 100.0% | |  | |  | | Percentage (%) | | 25.0% | 50.0% | 0.0% | 25.0% | 100.0% |  |
| Nursing | Frequency | 4 | 5 | | 0 | | 1 | | 10 | |  | | Nursing | | Frequency | | 4 | 2 | 2 | 2 | 10 |  |
|  | Percentage (%) | 40.0% | 50.0% | | 0.0% | | 10.0% | | 100.0% | |  | |  | | Percentage (%) | | 40.0% | 20.0% | 20.0% | 20.0% | 100.0% |  |
| Other | Frequency | 1 | 1 | | 0 | | 0 | | 2 | |  | | Other | | Frequency | | 1 | 0 | 1 | 0 | 2 |  |
|  | Percentage (%) | 50.0% | 50.0% | | 0.0% | | 0.0% | | 100.0% | |  | |  | | Percentage (%) | | 50.0% | 0.0% | 50.0% | 0.0% | 100.0% |  |
|  | Frequency | 52 | 32 | | 10 | | 8 | | 102 | |  | |  | | Frequency | | 34 | 39 | 21 | 8 | 102 |  |
|  | Percentage (%) | 51.0% | 31.4% | | 9.8% | | 7.8% | | 100.0% | |  | |  | | Percentage (%) | | 33.3% | 38.2% | 20.6% | 7.8% | 100.0% |  |
|  | Value | df | Sig. Asymptotic (bilateral) | | | | | |  | |  | |  | | Value | | df | Sig. Asymptotic (bilateral) | | |  |  |
| Pearson Chi-square | 22.005^a^ | 15 | 0.108 | |  | |  | |  | |  | | Pearson Chi-square | | 14.021^a^ | | 15 | 0.524 |  |  |  |  |
| Verisimilitude | 24.021 | 15 | 0.065 | |  | |  | |  | |  | | Verisimilitude | | 16.183 | | 15 | 0.370 |  |  |  |  |
| linear association | 1.658 | 1 | 0.198 | |  | |  | |  | |  | | linear association | | 0.662 | | 1 | 0.416 |  |  |  |  |
| N cases | 102 |  |  | |  | |  | |  | |  | | N cases | | 102 | |  |  |  |  |  |  |
| a. 18 cells (75.0%) have an expected frequency lower than 5. The expected minimum frequency is16. | | | | | | | | | | | | | | a. 18 cells (75.0%) have an expected frequency lower than 5. The expected minimum frequency is16. | | | | | | | | |
