# Supplementary material for: Prevalence of Stress in Healthcare Professionals during the COVID-19 Pandemic in Northeast Mexico: A Remote, Fast Survey Evaluation, Using an Adapted COVID-19 Stress Scales
Source: Int J Environ Res Public Health. 2020 Oct 19;17(20):7624. doi: 10.3390/ijerph17207624 (PMC7593933; doi:10.3390/ijerph17207624)
Supplement: Supplementary file 1 [file ijerph-17-07624-s001.zip › supp table/supp table 2.docx]

| **Medical / Nursing - CSS** |  |  |  | | | | Total |  | **Medical / Nursing - Danger + Contamination** | | |  | | | | Total |  |
| --- | --- | --- | --- | --- | --- | --- | --- | --- | --- | --- | --- | --- | --- | --- | --- | --- | --- |
|  |  |  | ABSENT | MILD | MODERATE | SEVERE |  |  |  |  |  | ABSENT | MILD | MODERATE | SEVERE |  |  |
| PROFESIONAL | Medical | Frequency | 9 | 53 | 24 | 5 | 91 |  | PROFESIONAL | Medical | Frequency | 6 | 23 | 49 | 13 | 91 |  |
|  |  | Percentage (%) | 9.9% | 58.2% | 26.4% | 5.5% | 100.0% |  |  |  | Percentage (%) | 6.6% | 25.3% | 53.8% | 14.3% | 100.0% |  |
|  | Nursing | Frequency | 0 | 3 | 1 | 0 | 4 |  |  | Nursing | Frequency | 0 | 2 | 2 | 0 | 4 |  |
|  |  | Percentage (%) | 0.0% | 75.0% | 25.0% | 0.0% | 100.0% |  |  |  | Percentage (%) | 0.0% | 50.0% | 50.0% | 0.0% | 100.0% |  |
| Total | | Frequency | 9 | 56 | 25 | 5 | 95 |  | Total | | Frequency | 6 | 25 | 51 | 13 | 95 |  |
|  |  | Percentage (%) | 9.5% | 58.9% | 26.3% | 5.3% | 100.0% |  |  |  | Percentage (%) | 6.3% | 26.3% | 53.7% | 13.7% | 100.0% |  |
|  | Value | df | Sig. Asymptotic (bilateral) | | |  |  |  |  | Value | df | Sig. Asymptotic (bilateral) | | |  |  |  |
| Pearson Chi-square | .801^a^ | 3 | 0.849 |  |  |  |  |  | Pearson Chi-square | 1.736^a^ | 3 | 0.629 |  |  |  |  |  |
| Verisimilitude | 1.376 | 3 | 0.711 |  |  |  |  |  | Verisimilitude | 2.356 | 3 | 0.502 |  |  |  |  |  |
| linear association | 0.025 | 1 | 0.873 |  |  |  |  |  | linear association | 0.161 | 1 | 0.688 |  |  |  |  |  |
| N cases | 95 |  |  |  |  |  |  |  | N cases | 95 |  |  |  |  |  |  |  |
| a. 5 cells (62.5%) have an expected frequency lower than 5. The expected minimum frequency is .21. | | | | | | | |  | a. 4 cells (50.0%) have an expected frequency lower than 5. The expected minimum frequency is .25. | | | | | | |  |  |
|  |  |  |  |  |  |  |  |  |  |  |  |  |  |  |  |  |  |
| **Medical / Nursing - Socioeconomical** | | |  |  |  |  |  |  | **Medical / Nursing - Xenophobia** | | |  | | | |  |  |
|  |  |  | ABSENT | MILD | MODERATE | SEVERE | Total |  |  |  |  | ABSENT | MILD | MODERATE | SEVERE | Total |  |
| PROFESIONAL | Medical | Frequency | 34 | 33 | 19 | 5 | 91 |  | PROFESIONAL | Medical | Frequency | 18 | 39 | 25 | 9 | 91 |  |
|  |  | Percentage (%) | 37.4% | 36.3% | 20.9% | 5.5% | 100.0% |  |  |  | Percentage (%) | 19.8% | 42.9% | 27.5% | 9.9% | 100.0% |  |
|  | Nursing | Frequency | 1 | 2 | 0 | 1 | 4 |  |  | Nursing | Frequency | 1 | 1 | 2 | 0 | 4 |  |
|  |  | Percentage (%) | 25.0% | 50.0% | 0.0% | 25.0% | 100.0% |  |  |  | Percentage (%) | 25.0% | 25.0% | 50.0% | 0.0% | 100.0% |  |
| Total |  | Frequency | 35 | 35 | 19 | 6 | 95 |  | Total | | Frequency | 19 | 40 | 27 | 9 | 95 |  |
|  |  | Percentage (%) | 36.8% | 36.8% | 20.0% | 6.3% | 100.0% |  |  |  | Percentage (%) | 20.0% | 42.1% | 28.4% | 9.5% | 100.0% |  |
|  | Value | df | Sig. Asymptotic (bilateral) | | |  |  |  |  | Value | df | Sig. Asymptotic (bilateral) | | |  |  |  |
| Pearson Chi-square | 3.498^a^ | 3 | 0.321 |  |  |  |  |  | Pearson Chi-square | 1.422^a^ | 3 | 0.700 |  |  |  |  |  |
| Verisimilitude | 3.349 | 3 | 0.341 |  |  |  |  |  | Verisimilitude | 1.723 | 3 | 0.632 |  |  |  |  |  |
| linear association | 0.396 | 1 | 0.529 |  |  |  |  |  | linear association | 0.015 | 1 | 0.901 |  |  |  |  |  |
| N cases | 95 |  |  |  |  |  |  |  | N cases | 95 |  |  |  |  |  |  |  |
| a. 4 cells (50.0%) have an expected frequency lower than 5. The expected minimum frequency is .25. | | | | | | | |  | a. 4 cells (50.0%) have an expected frequency lower than 5. The expected minimum frequency is .38. | | | | | | |  |  |
|  |  |  |  |  |  |  |  |  |  |  |  |  |  |  |  |  |  |
| **Medical / Nursing - Traumatic stress** | | |  | | | |  |  | **Medical / Nursing - Compulsive** | | |  | | | |  |  |
|  |  |  | ABSENT | MILD | MODERATE | SEVERE | Total |  |  |  |  | ABSENT | MILD | MODERATE | SEVERE | Total |  |
| PROFESIONAL | Medical | Frequency | 47 | 25 | 11 | 8 | 91 |  | PROFESIONAL | Medical | Frequency | 29 | 36 | 20 | 6 | 91 |  |
|  |  | Percentage (%) | 51.6% | 27.5% | 12.1% | 8.8% | 100.0% |  |  |  | Percentage (%) | 31.9% | 39.6% | 22.0% | 6.6% | 100.0% |  |
|  | Nursing | Frequency | 2 | 2 | 0 | 0 | 4 |  |  | Nursing | Frequency | 2 | 1 | 0 | 1 | 4 |  |
|  |  | Percentage (%) | 50.0% | 50.0% | 0.0% | 0.0% | 100.0% |  |  |  | Percentage (%) | 50.0% | 25.0% | 0.0% | 25.0% | 100.0% |  |
| Total | | Frequency | 49 | 27 | 11 | 8 | 95 |  | Total | | Frequency | 31 | 37 | 20 | 7 | 95 |  |
|  |  | Percentage (%) | 51.6% | 28.4% | 11.6% | 8.4% | 100.0% |  |  |  | Percentage (%) | 32.6% | 38.9% | 21.1% | 7.4% | 100.0% |  |
|  | Value | df | Sig. Asymptotic (bilateral) | | |  |  |  |  | Value | df | Sig. Asymptotic (bilateral) | | |  |  |  |
| Pearson Chi-square | 1.521^a^ | 3 | 0.677 |  |  |  |  |  | Pearson Chi-square | 3.235^a^ | 3 | 0.357 |  |  |  |  |  |
| Verisimilitude | 2.199 | 3 | 0.532 |  |  |  |  |  | Verisimilitude | 3.402 | 3 | 0.334 |  |  |  |  |  |
| linear association | 0.135 | 1 | 0.714 |  |  |  |  |  | linear association | 0.101 | 1 | 0.751 |  |  |  |  |  |
| N cases | 95 |  |  |  |  |  |  |  | N cases | 95 |  |  |  |  |  |  |  |
| a. 4 cells (50.0%) have an expected frequency lower than 5. The expected minimum frequency is .34. | | | | | | |  |  | a. 4 cells (50.0%) have an expected frequency lower than 5. The expected minimum frequency is .29. | | | | | | |  |  |
