# Supplementary material for: Prevalence of Stress in Healthcare Professionals during the COVID-19 Pandemic in Northeast Mexico: A Remote, Fast Survey Evaluation, Using an Adapted COVID-19 Stress Scales
Source: Int J Environ Res Public Health. 2020 Oct 19;17(20):7624. doi: 10.3390/ijerph17207624 (PMC7593933; doi:10.3390/ijerph17207624)
Supplement: Supplementary file 1 [file ijerph-17-07624-s001.zip › supp table/supp table 3.docx]

|  | **Work area - CSS** |  |  | | | | Total |  | **Work area - Danger + contamination** |  |  | | | | Total |
| --- | --- | --- | --- | --- | --- | --- | --- | --- | --- | --- | --- | --- | --- | --- | --- |
|  | Work area |  | ABSENT | MILD | MODERATE | SEVERE |  |  | Work area |  | ABSENT | MI[LD | MODERATE | SEVERE |  |
|  | Pediatrics | Frequency | 0 | 1 | 1 | 1 | 3 |  | Pediatrics | Frequency | 0 | 1 | 1 | 1 | 3 |
|  |  | Percentage (%) | 0.0% | 33.3% | 33.3% | 33.3% | 100.0% |  |  | Percentage (%) | 0.0% | 33.3% | 33.3% | 33.3% | 100.0% |
|  | First line healthcare provider | Frequency | 5 | 16 | 7 | 1 | 29 |  | First line healthcare provider | Frequency | 4 | 6 | 15 | 4 | 29 |
|  |  | Percentage (%) | 17.2% | 55.2% | 24.1% | 3.4% | 100.0% |  |  | Percentage (%) | 13.8% | 20.7% | 51.7% | 13.8% | 100.0% |
|  | COVID designated area | Frequency | 3 | 6 | 1 | 1 | 11 |  | COVID designated area | Frequency | 1 | 3 | 6 | 1 | 11 |
|  |  | Percentage (%) | 27.3% | 54.5% | 9.1% | 9.1% | 100.0% |  |  | Percentage (%) | 9.1% | 27.3% | 54.5% | 9.1% | 100.0% |
|  | Internal medicine | Frequency | 0 | 5 | 3 | 1 | 9 |  | Internal medicine | Frequency | 0 | 3 | 5 | 1 | 9 |
|  |  | Percentage (%) | 0.0% | 55.6% | 33.3% | 11.1% | 100.0% |  |  | Percentage (%) | 0.0% | 33.3% | 55.6% | 11.1% | 100.0% |
|  | Surgical | Frequency | 0 | 2 | 1 | 0 | 3 |  | Surgical | Frequency | 0 | 1 | 2 | 0 | 3 |
|  |  | Percentage (%) | 0.0% | 66.7% | 33.3% | 0.0% | 100.0% |  |  | Percentage (%) | 0.0% | 33.3% | 66.7% | 0.0% | 100.0% |
|  | ICU | Frequency | 0 | 4 | 2 | 0 | 6 |  | ICU | Frequency | 0 | 1 | 4 | 1 | 6 |
|  |  | Percentage (%) | 0.0% | 66.7% | 33.3% | 0.0% | 100.0% |  |  | Percentage (%) | 0.0% | 16.7% | 66.7% | 16.7% | 100.0% |
|  | Radiology | Frequency | 0 | 2 | 0 | 0 | 2 |  | Radiology | Frequency | 0 | 1 | 1 | 0 | 2 |
|  |  | Percentage (%) | 0.0% | 100.0% | 0.0% | 0.0% | 100.0% |  |  | Percentage (%) | 0.0% | 50.0% | 50.0% | 0.0% | 100.0% |
|  | OBGYN | Frequency | 0 | 4 | 7 | 0 | 11 |  | OBGYN | Frequency | 0 | 2 | 6 | 3 | 11 |
|  |  | Percentage (%) | 0.0% | 36.4% | 63.6% | 0.0% | 100.0% |  |  | Percentage (%) | 0.0% | 18.2% | 54.5% | 27.3% | 100.0% |
|  | ER | Frequency | 0 | 5 | 4 | 0 | 9 |  | ER | Frequency | 1 | 2 | 5 | 1 | 9 |
|  |  | Percentage (%) | 0.0% | 55.6% | 44.4% | 0.0% | 100.0% |  |  | Percentage (%) | 11.1% | 22.2% | 55.6% | 11.1% | 100.0% |
|  | Others | Frequency | 1 | 15 | 3 | 1 | 20 |  | Others | Frequency | 1 | 6 | 11 | 2 | 20 |
|  |  | Percentage (%) | 5.0% | 75.0% | 15.0% | 5.0% | 100.0% |  |  | Percentage (%) | 5.0% | 30.0% | 55.0% | 10.0% | 100.0% |
|  | Total | Frequency | 9 | 60 | 29 | 5 | 103 |  | Total | Frequency | 7 | 26 | 56 | 14 | 103 |
|  |  | Percentage (%) | 8.7% | 58.3% | 28.2% | 4.9% | 100.0% |  |  | Percentage (%) | 6.8% | 25.2% | 54.4% | 13.6% | 100.0% |
|  |  | Value | df | Sig. Asymptotic (bilateral) | | |  |  |  | Value | df | Sig. Asymptotic (bilateral) | | |  |
|  | Pearson Chi-square | 30.907^a^ | 27 | 0.275 |  |  |  |  | Pearson Chi-square | 10.639^a^ | 27 | 0.998 |  |  |  |
|  | Verisimilitude | 30.886 | 27 | 0.276 |  |  |  |  | Verisimilitude | 12.485 | 27 | 0.992 |  |  |  |
|  | linear association | 1.495 | 1 | 0.221 |  |  |  |  | linear association | 0.391 | 1 | 0.532 |  |  |  |
|  | N cases | 103 |  |  |  |  |  |  | N cases | 103 |  |  |  |  |  |
|  | a. 32 cells (80.0%) have an expected frequency lower than 5. The expected minimum frequency is .10. | | | | | | |  | a. 34 cells (85.0%) have an expected frequency lower than 5. The expected minimum frequency is .14. | | | |  |  |  |
|  | | | | | | | |  |  |  |  |  |  |  |  |
|  | **Work area - Socioeconomical** |  |  | | | | Total |  | **Work area - Xenophobia** |  |  |  |  |  |  |
|  | Work area |  | ABSENT | MILD | MODERATE | SEVERE |  |  | Work area - Xenophobia |  | ABSENT | MILD | MODERATE | SEVERE | Total |
|  | Pediatrics | Frequency | 1 | 0 | 1 | 1 | 3 |  | Pediatrics | Frequency | 0 | 0 | 3 | 0 | 3 |
|  |  | Percentage (%) | 33.3% | 0.0% | 33.3% | 33.3% | 100.0% |  |  | Percentage (%) | 0.0% | 0.0% | 100.0% | 0.0% | 100.0% |
|  | First line healthcare provider | Frequency | 11 | 10 | 7 | 1 | 29 |  | First line healthcare provider | Frequency | 10 | 9 | 8 | 2 | 29 |
|  |  | Percentage (%) | 37.9% | 34.5% | 24.1% | 3.4% | 100.0% |  |  | Percentage (%) | 34.5% | 31.0% | 27.6% | 6.9% | 100.0% |
|  | COVID designated area | Frequency | 7 | 3 | 1 | 0 | 11 |  | COVID designated area | Frequency | 2 | 8 | 0 | 1 | 11 |
|  |  | Percentage (%) | 63.6% | 27.3% | 9.1% | 0.0% | 100.0% |  |  | Percentage (%) | 18.2% | 72.7% | 0.0% | 9.1% | 100.0% |
|  | Internal medicine | Frequency | 2 | 6 | 1 | 0 | 9 |  | Internal medicine | Frequency | 2 | 2 | 4 | 1 | 9 |
|  |  | Percentage (%) | 22.2% | 66.7% | 11.1% | 0.0% | 100.0% |  |  | Percentage (%) | 22.2% | 22.2% | 44.4% | 11.1% | 100.0% |
|  | Surgical | Frequency | 1 | 1 | 0 | 1 | 3 |  | Surgical | Frequency | 0 | 1 | 2 | 0 | 3 |
|  |  | Percentage (%) | 33.3% | 33.3% | 0.0% | 33.3% | 100.0% |  |  | Percentage (%) | 0.0% | 33.3% | 66.7% | 0.0% | 100.0% |
|  | ICU | Frequency | 2 | 2 | 2 | 0 | 6 |  | ICU | Frequency | 0 | 2 |  | 0 | 6 |
|  |  | Percentage (%) | 33.3% | 33.3% | 33.3% | 0.0% | 100.0% |  |  | Percentage (%) | 0.0% | 33.3% | 66.7% | 0.0% | 100.0% |
|  | Radiology | Frequency | 0 | 2 | 0 | 0 | 2 |  | Radiology | Frequency | 1 | 1 | 0 | 0 | 2 |
|  |  | Percentage (%) | 0.0% | 100.0% | 0.0% | 0.0% | 100.0% |  |  | Percentage (%) | 50.0% | 50.0% | 0.0% | 0.0% | 100.0% |
|  | OBGYN | Frequency | 2 | 4 | 3 | 2 | 11 |  | OBGYN | Frequency | 2 | 3 | 3 | 3 | 11 |
|  |  | Percentage (%) | 18.2% | 36.4% | 27.3% | 18.2% | 100.0% |  |  | Percentage (%) | 18.2% | 27.3% | 27.3% | 27.3% | 100.0% |
|  | ER | Frequency | 2 | 3 | 3 | 1 | 9 |  | ER | Frequency | 3 | 3 | 2 | 1 | 9 |
|  |  | Percentage (%) | 22.2% | 33.3% | 33.3% | 11.1% | 100.0% |  |  | Percentage (%) | 33.3% | 33.3% | 22.2% | 11.1% | 100.0% |
|  | Others | Frequency | 9 | 6 | 4 | 1 | 20 |  | Others | Frequency | 1 | 14 | 3 | 2 | 20 |
|  |  | Percentage (%) | 45.0% | 30.0% | 20.0% | 5.0% | 100.0% |  |  | Percentage (%) | 5.0% | 70.0% | 15.0% | 10.0% | 100.0% |
|  | Total | Frequency | 37 | 37 | 22 | 7 | 103 |  | Total | Frequency | 21 | 43 | 29 | 10 | 103 |
|  |  | Percentage (%) | 35.9% | 35.9% | 21.4% | 6.8% | 100.0% |  |  | Percentage (%) | 20.4% | 41.7% | 28.2% | 9.7% | 100.0% |
|  |  | Value | df | Sig. Asymptotic (bilateral) | | |  |  |  | Value | df | Sig. Asymptotic (bilateral) | | |  |
|  | Pearson Chi-square | 26.530^a^ | 27 | 0.489 |  |  |  |  | Pearson Chi-square | 40.607^a^ | 27 | 0.045 |  |  |  |
|  | Verisimilitude | 26.464 | 27 | 0.493 |  |  |  |  | Verisimilitude | 44.182 | 27 | 0.020 |  |  |  |
|  | linear association | 0.554 | 1 | 0.457 |  |  |  |  | linear association | 1.111 | 1 | 0.292 |  |  |  |
|  | N cases | 103 |  |  |  |  |  |  | N cases | 103 |  |  |  |  |  |
|  | a. 35 cells (87.5%) have an expected frequency lower than 5. The expected minimum frequency is .14. | | | |  |  |  |  | a. 35 cells (87.5%) have an expected frequency lower than 5. The expected minimum frequency is .19. | | | |  |  |  |

|  | **Work area - Traumatic stress** |  |  | | | |  |  | **Work area - Compulsive** |  |  |  |  |  |  |
| --- | --- | --- | --- | --- | --- | --- | --- | --- | --- | --- | --- | --- | --- | --- | --- |
|  | Work area |  | ABSENT | MILD | MODERATE | SEVERE | Total |  | Work area |  | ABSENT | MILD | MODERATE | SEVERE | Total |
|  | Pediatrics | Frequency | 2 | 0 | 0 | 1 | 3 |  | Pediatrics | Frequency | 2 | 0 | 0 | 1 | 3 |
|  |  | Percentage (%) | 66.7% | 0.0% | 0.0% | 33.3% | 100.0% |  |  | Percentage (%) | 66.7% | 0.0% | 0.0% | 33.3% | 100.0% |
|  | First line healthcare provider | Frequency | 15 | 9 | 4 | 1 | 29 |  | First line healthcare provider | Frequency | 9 | 15 | 5 | 0 | 29 |
|  |  | Percentage (%) | 51.7% | 31.0% | 13.8% | 3.4% | 100.0% |  |  | Percentage (%) | 31.0% | 51.7% | 17.2% | 0.0% | 100.0% |
|  | COVID designated area | Frequency | 5 | 5 | 1 | 0 | 11 |  | COVID designated area | Frequency | 6 | 2 | 2 | 1 | 11 |
|  |  | Percentage (%) | 45.5% | 45.5% | 9.1% | 0.0% | 100.0% |  |  | Percentage (%) | 54.5% | 18.2% | 18.2% | 9.1% | 100.0% |
|  | Internal medicine | Frequency | 3 | 3 | 1 | 2 | 9 |  | Internal medicine | Frequency | 2 | 1 | 4 | 2 | 9 |
|  |  | Percentage (%) | 33.3% | 33.3% | 11.1% | 22.2% | 100.0% |  |  | Percentage (%) | 22.2% | 11.1% | 44.4% | 22.2% | 100.0% |
|  | Surgical | Frequency | 1 | 2 | 0 | 0 | 3 |  | Surgical | Frequency | 1 | 1 | 0 | 1 | 3 |
|  |  | Percentage (%) | 33.3% | 66.7% | 0.0% | 0.0% | 100.0% |  |  | Percentage (%) | 33.3% | 33.3% | 0.0% | 33.3% | 100.0% |
|  | ICU | Frequency | 4 | 2 | 0 | 0 | 6 |  | ICU | Frequency | 1 | 3 | 2 | 0 | 6 |
|  |  | Percentage (%) | 66.7% | 33.3% | 0.0% | 0.0% | 100.0% |  |  | Percentage (%) | 16.7% | 50.0% | 33.3% | 0.0% | 100.0% |
|  | Radiology | Frequency | 1 | 1 | 0 | 0 | 2 |  | Radiology | Frequency | 0 | 1 | 1 | 0 | 2 |
|  |  | Percentage (%) | 50.0% | 50.0% | 0.0% | 0.0% | 100.0% |  |  | Percentage (%) | 0.0% | 50.0% | 50.0% | 0.0% | 100.0% |
|  | OBGYN | Frequency | 4 | 1 | 2 | 4 | 11 |  | OBGYN | Frequency | 1 | 5 | 4 | 1 | 11 |
|  |  | Percentage (%) | 36.4% | 9.1% | 18.2% | 36.4% | 100.0% |  |  | Percentage (%) | 9.1% | 45.5% | 36.4% | 9.1% | 100.0% |
|  | ER | Frequency | 4 | 5 | 0 | 0 | 9 |  | ER | Frequency | 4 | 3 | 1 | 1 | 9 |
|  |  | Percentage (%) | 44.4% | 55.6% | 0.0% | 0.0% | 100.0% |  |  | Percentage (%) | 44.4% | 33.3% | 11.1% | 11.1% | 100.0% |
|  | Others | Frequency | 13 | 4 | 3 | 0 | 20 |  | Others | Frequency | 8 | 9 | 2 | 1 | 20 |
|  |  | Percentage (%) | 65.0% | 20.0% | 15.0% | 0.0% | 100.0% |  |  | Percentage (%) | 40.0% | 45.0% | 10.0% | 5.0% | 100.0% |
|  | Total | Frequency | 52 | 32 | 11 | 8 | 103 |  | Total | Frequency | 34 | 40 | 21 | 8 | 103 |
|  |  | Percentage (%) | 50.5% | 31.1% | 10.7% | 7.8% | 100.0% |  |  | Percentage (%) | 33.0% | 38.8% | 20.4% | 7.8% | 100.0% |
|  |  | Value | df | Sig. Asymptotic (bilateral) | | |  |  |  | Value | df | Sig. Asymptotic (bilateral) | | |  |
|  | Pearson Chi-square | 34.806^a^ | 27 | 0.144 |  |  |  |  | Pearson Chi-square | 31.531^a^ | 27 | 0.250 |  |  |  |
|  | Verisimilitude | 34.739 | 27 | 0.146 |  |  |  |  | Verisimilitude | 34.678 | 27 | 0.147 |  |  |  |
|  | linear association | 0.145 | 1 | 0.703 |  |  |  |  | linear association | 0.031 | 1 | 0.860 |  |  |  |
|  | N cases | 103 |  |  |  |  |  |  | N cases | 103 |  |  |  |  |  |
|  | a. 34 cells (85.0%) have an expected frequency lower than 5. The expected minimum frequency is .16. | | | | | | |  | a. 35 cells (87.5%) have an expected frequency lower than 5. The expected minimum frequency is .16. | | | | | | |
