# Supplementary material for: Prevalence of Stress in Healthcare Professionals during the COVID-19 Pandemic in Northeast Mexico: A Remote, Fast Survey Evaluation, Using an Adapted COVID-19 Stress Scales
Source: Int J Environ Res Public Health. 2020 Oct 19;17(20):7624. doi: 10.3390/ijerph17207624 (PMC7593933; doi:10.3390/ijerph17207624)
Supplement: Supplementary file 1 [file ijerph-17-07624-s001.zip › supp table/supp table 4.docx]

| **Diagnosis - CSS** |  |  |  |  |  |  |  |  | | **Diagnosis - Danger + Contamination** | | | | |  |  |  |  |  | |  | |  |
| --- | --- | --- | --- | --- | --- | --- | --- | --- | --- | --- | --- | --- | --- | --- | --- | --- | --- | --- | --- | --- | --- | --- | --- |
|  |  |  | ABSENT | MILD | MODERETE | SEVERE | Total |  | |  | |  |  | ABSENT | MILD | MODERETE | SEVERE | Total |  | |  | |  |
| DIAGNOSIS | Yes | Frequency | 1 | 2 | 2 | 0 | 5 |  | | DIAGNOSIS | | Yes | Frequency | 1 | 1 | 3 | 0 | 5 |  | |  | |  |
|  |  | Percentage (%) | 20.0% | 40.0% | 40.0% | 0.0% | 100.0% |  | |  | |  | Percentage (%) | 20.0% | 20.0% | 60.0% | 0.0% | 100.0% |  | |  | |  |
|  | No | Frequency | 8 | 58 | 27 | 5 | 98 |  | |  | | No | Frequency | 6 | 25 | 53 | 14 | 98 |  | |  | |  |
|  |  | Percentage (%) | 8.2% | 59.2% | 27.6% | 5.1% | 100.0% |  | |  | |  | Percentage (%) | 6.1% | 25.5% | 54.1% | 14.3% | 100.0% |  | |  | |  |
| Total |  | Frequency | 9 | 60 | 29 | 5 | 103 |  | | Total | |  | Frequency | 7 | 26 | 56 | 14 | 103 |  | |  | |  |
|  |  | Percentage (%) | 8.7% | 58.3% | 28.2% | 4.9% | 100.0% |  | |  | |  | Percentage (%) | 6.8% | 25.2% | 54.4% | 13.6% | 100.0% |  | |  | |  |
|  | Value | df | Sig. Asymptotic (bilateral) | | |  |  |  | |  | | Value | df | Sig. Asymptotic (bilateral) | | |  |  |  | |  | |  |
| Pearson Chi-square | 1.580^a^ | 3 | 0.664 |  |  |  |  |  | | Pearson Chi-square | | 2.150^a^ | 3 | 0.542 |  |  |  |  |  | |  | |  |
| Verisimilitude | 1.634 | 3 | 0.652 |  |  |  |  |  | | Verisimilitude | | 2.391 | 3 | 0.495 |  |  |  |  |  | |  | |  |
| linear association | 0.276 | 1 | 0.599 |  |  |  |  |  | | linear association | | 1.334 | 1 | 0.248 |  |  |  |  |  | |  | |  |
| N cases | 103 |  |  |  |  |  |  |  | | N cases | | 103 |  |  |  |  |  |  |  | |  | |  |
| a. 5 cells (62.5%) have an expected frequency lower than 5. The minimum expected frequency is es .24. | | | | | | | | |  | | a. 4 cells (50.0%) have an expected frequency lower than 5. The minimum expected frequency is es .34. | | | | | | | | | | | | |
|  |  |  |  |  |  |  |  |  | |  | |  |  |  |  |  |  |  |  | |  | |  |
| **Diagnosis - Socialeconomical** | |  |  |  |  |  |  |  | | **Diagnosis - Xenophobia** | | |  |  |  |  |  |  |  | |  | |  |
|  |  |  | ABSENT | MILD | MODERETE | SEVERE | Total |  | |  | |  |  | ABSENT | MILD | MODERETE | SEVERE | Total |  | |  | |  |
| DIAGNOSIS | Yes | Frequency | 1 | 3 | 1 | 0 | 5 |  | | Diagnosis | | Yes | Frequency | 2 | 2 | 1 | 0 | 5 |  | |  | |  |
|  |  | Percentage (%) | 20.0% | 60.0% | 20.0% | 0.0% | 100.0% |  | |  | |  | Percentage (%) | 40.0% | 40.0% | 20.0% | 0.0% | 100.0% |  | |  | |  |
|  | No | Frequency | 36 | 34 | 21 | 7 | 98 |  | |  | | No | Frequency | 19 | 41 | 28 | 10 | 98 |  | |  | |  |
|  |  | Percentage (%) | 36.7% | 34.7% | 21.4% | 7.1% | 100.0% |  | |  | |  | Percentage (%) | 19.4% | 41.8% | 28.6% | 10.2% | 100.0% |  | |  | |  |
| Total |  | Frequency | 37 | 37 | 22 | 7 | 103 |  | | Total | |  | Frequency | 21 | 43 | 29 | 10 | 103 |  | |  | |  |
|  |  | Percentage (%) | 35.9% | 35.9% | 21.4% | 6.8% | 100.0% |  | |  | |  | Percentage (%) | 20.4% | 41.7% | 28.2% | 9.7% | 100.0% |  | |  | |  |
|  | Value | df | Sig. Asymptotic (bilateral) | | |  |  |  | |  | | Value | df | Sig. Asymptotic (bilateral) | | |  |  |  | |  | |  |
| Pearson Chi-square | 1.581^a^ | 3 | 0.664 |  |  |  |  |  | | Pearson Chi-square | | 1.630^a^ | 3 | 0.653 |  |  |  |  |  | |  | |  |
| Verisimilitude | 1.852 | 3 | 0.604 |  |  |  |  |  | | Verisimilitude | | 1.920 | 3 | 0.589 |  |  |  |  |  | |  | |  |
| linear association | 0.083 | 1 | 0.773 |  |  |  |  |  | | linear association | | 1.568 | 1 | 0.210 |  |  |  |  |  | |  | |  |
| N cases | 103 |  |  |  |  |  |  |  | | N cases | | 103 |  |  |  |  |  |  |  | |  | |  |
| a. 4 cells (50.0%) have an expected frequency lower than 5. The minimum expected frequency is es .34. | | | | | | | | |  | | a. 4 cells (50.0%) have an expected frequency lower than 5. The minimum expected frequency is es .49. | | | | | | | | |  | |  | |
|  |  |  |  |  |  |  |  |  | |  | |  |  |  |  |  |  |  |  | |  | |  |
| **Diagnosis - Traumatic stress** | |  |  |  |  |  |  |  | | **Diagnosis - Compulsive** | | |  |  |  |  |  |  |  | |  | |  |
|  |  |  | ABSENT | MILD | MODERETE | SEVERE | Total |  | |  | |  |  | ABSENT | MILD | MODERETE | SEVERE | Total |  | |  | |  |
| DIAGNOSIS | Yes | Frequency | 2 | 2 | 1 | 0 | 5 |  | | DIAGNOSIS | | Yes | Frequency | 2 | 1 | 1 | 1 | 5 |  | |  | |  |
|  |  | Percentage (%) | 40.0% | 40.0% | 20.0% | 0.0% | 100.0% |  | |  | |  | Percentage (%) | 40.0% | 20.0% | 20.0% | 20.0% | 100.0% |  | |  | |  |
|  | No | Frequency | 50 | 30 | 10 | 8 | 98 |  | |  | | No | Frequency | 32 | 39 | 20 | 7 | 98 |  | |  | |  |
|  |  | Percentage (%) | 51.0% | 30.6% | 10.2% | 8.2% | 100.0% |  | |  | |  | Percentage (%) | 32.7% | 39.8% | 20.4% | 7.1% | 100.0% |  | |  | |  |
| Total |  | Frequency | 52 | 32 | 11 | 8 | 103 |  | | Total | |  | Frequency | 34 | 40 | 21 | 8 | 103 |  | |  | |  |
|  |  | Percentage (%) | 50.5% | 31.1% | 10.7% | 7.8% | 100.0% |  | |  | |  | Percentage (%) | 33.0% | 38.8% | 20.4% | 7.8% | 100.0% |  | |  | |  |
|  | Value | df | Sig. Asymptotic (bilateral) | | |  |  |  | |  | | Value | df | Sig. Asymptotic (bilateral) | | |  |  |  | |  | |  |
| Pearson Chi-square | 1.085^a^ | 3 | 0.781 |  |  |  |  |  | | Pearson Chi-square | | 1.571^a^ | 3 | 0.666 |  |  |  |  |  | |  | |  |
| Verisimilitude | 1.387 | 3 | 0.709 |  |  |  |  |  | | Verisimilitude | | 1.372 | 3 | 0.712 |  |  |  |  |  | |  | |  |
| linear association | 0.060 | 1 | 0.806 |  |  |  |  |  | | linear association | | 0.030 | 1 | 0.862 |  |  |  |  |  | |  | |  |
| N cases | 103 |  |  |  |  |  |  |  | | N cases | | 103 |  |  |  |  |  |  |  | |  | |  |
| a. 4 cells (50.0%) have an expected frequency lower than 5. The minimum expected frequency is es .39. | | | | | | | | |  | | a. 4 cells (50.0%) have an expected frequency lower than 5. The minimum expected frequency is es .39. | | | | | | | | |  | |  | |
