# Supplementary material for: Prevalence of Stress in Healthcare Professionals during the COVID-19 Pandemic in Northeast Mexico: A Remote, Fast Survey Evaluation, Using an Adapted COVID-19 Stress Scales
Source: Int J Environ Res Public Health. 2020 Oct 19;17(20):7624. doi: 10.3390/ijerph17207624 (PMC7593933; doi:10.3390/ijerph17207624)
Supplement: Supplementary file 1 [file ijerph-17-07624-s001.zip › supp table/supp table 5.docx]

| **Work with COVID patients - CSS** | | | | |  |  |  |  | **Work with COVID patients - Danger + Contamination** | | | |  |  |  |  |  |
| --- | --- | --- | --- | --- | --- | --- | --- | --- | --- | --- | --- | --- | --- | --- | --- | --- | --- |
|  |  |  | ABSENT | MILD | MODERETE | SEVERE | Total |  |  |  |  | ABSENT | MILD | MODERATE | SEVERE | Total |  |
| Work with  COVID patients | Yes | Frequency | 4 | 32 | 20 | 4 | 60 |  | Work with  COVID patients | Yes | Frequency | 4 | 12 | 33 | 11 | 60 |  |
|  |  | Percentage (%) | 6.7% | 53.3% | 33.3% | 6.7% | 100.0% |  |  |  | Percentage (%) | 6.7% | 20.0% | 55.0% | 18.3% | 100.0% |  |
|  | No | Frequency | 5 | 28 | 9 | 1 | 43 |  |  | No | Frequency | 3 | 14 | 23 | 3 | 43 |  |
|  |  | Percentage (%) | 11.6% | 65.1% | 20.9% | 2.3% | 100.0% |  |  |  | Percentage (%) | 7.0% | 32.6% | 53.5% | 7.0% | 100.0% |  |
| Total |  | Frequency | 9 | 60 | 29 | 5 | 103 |  | Total |  | Frequency | 7 | 26 | 56 | 14 | 103 |  |
|  |  | Percentage (%) | 8.7% | 58.3% | 28.2% | 4.9% | 100.0% |  |  |  | Percentage (%) | 6.8% | 25.2% | 54.4% | 13.6% | 100.0% |  |
|  | Value | df | Sig. Asymptotic (bilateral) | | |  |  |  |  | Value | df | Sig. Asymptotic (bilateral) | | |  |  |  |
| Pearson Chi-square | 3.644^a^ | 3 | 0.303 |  |  |  |  |  | Pearson Chi-square | 3.956^a^ | 3 | 0.266 |  |  |  |  |  |
| Verisimilitude | 3.766 | 3 | 0.288 |  |  |  |  |  | Verisimilitude | 4.134 | 3 | 0.247 |  |  |  |  |  |
| linear association | 3.043 | 1 | 0.081 |  |  |  |  |  | linear association | 1.706 | 1 | 0.191 |  |  |  |  |  |
| N cases | 103 |  |  |  |  |  |  |  | N cases | 103 |  |  |  |  |  |  |  |
| a. 3 cells (37.5%) have an expected Frequency lower than 5. The minimum expected frequency is 2.09. | | | | | | | | | a. 2 cells (25.0%) have an expected Frequency lower than 5. The minimum expected frequency is 2.92. | | | | | | | | |
|  |  |  |  |  |  |  |  |  |  |  |  |  |  |  |  |  |  |
| **Work with COVID patients - Socioeconomical** | | |  |  |  |  |  |  | **Work with COVID patients - Xenophobia** | | |  |  |  |  |  |  |
|  |  |  | ABSENT | MILD | MODERETE | SEVERE | Total |  |  |  |  | ABSENT | MILD | MODERETE | SEVERE | Total |  |
| Work with  COVID patients | Yes | Frequency | 20 | 21 | 14 | 5 | 60 |  | Work with  COVID patients | Yes | Frequency | 12 | 22 | 19 | 7 | 60 |  |
|  |  | Percentage (%) | 33.3% | 35.0% | 23.3% | 8.3% | 100.0% |  |  |  | Percentage (%) | 20.0% | 36.7% | 31.7% | 11.7% | 100.0% |  |
|  | No | Frequency | 17 | 16 | 8 | 2 | 43 |  |  | No | Frequency | 9 | 21 | 10 | 3 | 43 |  |
|  |  | Percentage (%) | 39.5% | 37.2% | 18.6% | 4.7% | 100.0% |  |  |  | Percentage (%) | 20.9% | 48.8% | 23.3% | 7.0% | 100.0% |  |
| Total |  | Frequency | 37 | 37 | 22 | 7 | 103 |  | Total |  | Frequency | 21 | 43 | 29 | 10 | 103 |  |
|  |  | Percentage (%) | 35.9% | 35.9% | 21.4% | 6.8% | 100.0% |  |  |  | Percentage (%) | 20.4% | 41.7% | 28.2% | 9.7% | 100.0% |  |
|  | Value | df | Sig. Asymptotic (bilateral) | | |  |  |  |  | Value | df | Sig. Asymptotic (bilateral) | | |  |  |  |
| Pearson Chi-square | 1.064^a^ | 3 | 0.786 |  |  |  |  |  | Pearson Chi-square | 2.096^a^ | 3 | 0.553 |  |  |  |  |  |
| Verisimilitude | 1.088 | 3 | 0.780 |  |  |  |  |  | Verisimilitude | 2.120 | 3 | 0.548 |  |  |  |  |  |
| linear association | 0.833 | 1 | 0.361 |  |  |  |  |  | linear association | 0.647 | 1 | 0.421 |  |  |  |  |  |
| N cases | 103 |  |  |  |  |  |  |  | N cases | 103 |  |  |  |  |  |  |  |
| a. 2 cells (25.0%) have an expected Frequency lower than 5. The minimum expected frequency is 2.92. | | | | | | | | | a. 1 cells (12.5%) have an expected Frequency lower than 5. The minimum expected frequency is 4.17. | | | | | | | | |
|  |  |  |  |  |  |  |  |  |  |  |  |  |  |  |  |  |  |
| **Work with COVID patients - Traumatic stress** | | |  |  |  |  |  |  | **Work with COVID patients - Compulsive** | | |  |  |  |  |  |  |
|  |  |  | ABSENT | MILD | MODERETE | SEVERE | Total |  |  |  |  | ABSENT | MILD | MODERETE | SEVERE | Total |  |
| Work with  COVID patients | Yes | Frequency | 21 | 27 | 7 | 5 | 60 |  | Work with  COVID patients | Yes | Frequency | 17 | 21 | 15 | 7 | 60 |  |
|  |  | Percentage (%) | 35.0% | 45.0% | 11.7% | 8.3% | 100.0% |  |  |  | Percentage (%) | 28.3% | 35.0% | 25.0% | 11.7% | 100.0% |  |
|  | No | Frequency | 31 | 5 | 4 | 3 | 43 |  |  | No | Frequency | 17 | 19 | 6 | 1 | 43 |  |
|  |  | Percentage (%) | 72.1% | 11.6% | 9.3% | 7.0% | 100.0% |  |  |  | Percentage (%) | 39.5% | 44.2% | 14.0% | 2.3% | 100.0% |  |
| Total |  | Frequency | 52 | 32 | 11 | 8 | 103 |  | Total |  | Frequency | 34 | 40 | 21 | 8 | 103 |  |
|  |  | Percentage (%) | 50.5% | 31.1% | 10.7% | 7.8% | 100.0% |  |  |  | Percentage (%) | 33.0% | 38.8% | 20.4% | 7.8% | 100.0% |  |
|  | Value | df | Sig. Asymptotic (bilateral) | | |  |  |  |  | Value | df | Sig. Asymptotic (bilateral) | | |  |  |  |
| Pearson Chi-square | 15.996^a^ | 3 | 0.001 |  |  |  |  |  | Pearson Chi-square | 5.810^a^ | 3 | 0.121 |  |  |  |  |  |
| Verisimilitude | 17.074 | 3 | 0.001 |  |  |  |  |  | Verisimilitude | 6.328 | 3 | 0.097 |  |  |  |  |  |
| linear association | 8.298 | 1 | 0.004 |  |  |  |  |  | linear association | 3.865 | 1 | 0.049 |  |  |  |  |  |
| N cases | 103 |  |  |  |  |  |  |  | N cases | 103 |  |  |  |  |  |  |  |
| a. 3 cells (37.5%) have an expected Frequency lower than 5. The minimum expected frequency is 3.34. | | | | | | | | | a. 2 cells (25.0%) have an expected Frequency lower than 5. The minimum expected frequency is 3.34. | | | | | | | | |
