# Supplementary material for: Prevalence of Stress in Healthcare Professionals during the COVID-19 Pandemic in Northeast Mexico: A Remote, Fast Survey Evaluation, Using an Adapted COVID-19 Stress Scales
Source: Int J Environ Res Public Health. 2020 Oct 19;17(20):7624. doi: 10.3390/ijerph17207624 (PMC7593933; doi:10.3390/ijerph17207624)
Supplement: Supplementary file 1 [file ijerph-17-07624-s001.zip › supp table/supp table 6.docx]

| **# Patients - CSS** | |  |  |  |  |  |  |  | **# of Patients - Danger + Contamination** | | |  |  |  |  |  |  |
| --- | --- | --- | --- | --- | --- | --- | --- | --- | --- | --- | --- | --- | --- | --- | --- | --- | --- |
|  |  |  | ABSENT | MILD | MODERATE | SEVERE | Total |  |  |  |  | ABSENT | MILD | MODERATE | SEVERE | Total |  |
| # of Patients | 0 | Frequency | 5 | 28 | 9 | 1 | 43 |  | # of Patients | 0 | Frequency | 3 | 14 | 23 | 3 | 43 |  |
|  |  | Percentage (%) | 11.6% | 65.1% | 20.9% | 2.3% | 100.0% |  |  |  | Percentage (%) | 7.0% | 32.6% | 53.5% | 7.0% | 100.0% |  |
|  | 1 to 5 | Frequency | 1 | 17 | 13 | 2 | 33 |  |  | 1 to 5 | Frequency | 1 | 7 | 18 | 7 | 33 |  |
|  |  | Percentage (%) | 3.0% | 51.5% | 39.4% | 6.1% | 100.0% |  |  |  | Percentage (%) | 3.0% | 21.2% | 54.5% | 21.2% | 100.0% |  |
|  | 5 to 10 | Frequency | 1 | 4 | 2 | 2 | 9 |  |  | 5 to 10 | Frequency | 0 | 2 | 4 | 3 | 9 |  |
|  |  | Percentage (%) | 11.1% | 44.4% | 22.2% | 22.2% | 100.0% |  |  |  | Percentage (%) | 0.0% | 22.2% | 44.4% | 33.3% | 100.0% |  |
|  | 10 to 20 | Frequency | 0 | 2 | 4 | 0 | 6 |  |  | 10 to 20 | Frequency | 0 | 2 | 3 | 1 | 6 |  |
|  |  | Percentage (%) | 0.0% | 33.3% | 66.7% | 0.0% | 100.0% |  |  |  | Percentage (%) | 0.0% | 33.3% | 50.0% | 16.7% | 100.0% |  |
|  | > 20 | Frequency | 2 | 9 | 1 | 0 | 12 |  |  | > 20 | Frequency | 3 | 1 | 8 | 0 | 12 |  |
|  |  | Percentage (%) | 16.7% | 75.0% | 8.3% | 0.0% | 100.0% |  |  |  | Percentage (%) | 25.0% | 8.3% | 66.7% | 0.0% | 100.0% |  |
| Total |  | Frequency | 9 | 60 | 29 | 5 | 103 |  | Total |  | Frequency | 7 | 26 | 56 | 14 | 103 |  |
|  |  | Percentage (%) | 8.7% | 58.3% | 28.2% | 4.9% | 100.0% |  |  |  | Percentage (%) | 6.8% | 25.2% | 54.4% | 13.6% | 100.0% |  |
|  | Value | df | Sig. Asymptotic (bilateral) | | |  |  |  |  | Value | df | Sig. Asymptotic (bilateral) | | |  |  |  |
| Pearson Chi-square | 19.561^a^ | 12 | 0.076 |  |  |  |  |  | Pearson Chi-square | 17.804^a^ | 12 | 0.122 |  |  |  |  |  |
| Verisimilitude | 18.423 | 12 | 0.103 |  |  |  |  |  | Verisimilitude | 17.966 | 12 | 0.117 |  |  |  |  |  |
| N cases | 103 |  |  |  |  |  |  |  | N cases | 103 |  |  |  |  |  |  |  |
| a. 14 cells (70.0%) have an expected Frequency lower than 5. The expected minimum frequency is .29. | | | | | | | | | a. 14 cells (70.0%) have an expected Frequency lower than 5. The expected minimum frequency is .41. | | | | | | | | |
|  |  |  |  |  |  |  |  |  |  |  |  |  |  |  |  |  |  |
| **# Patients - Socioeconomical** | | |  |  |  |  |  |  | **# Patients - Xenophobia** | | |  |  |  |  |  |  |
|  |  |  | ABSENT | MILD | MODERATE | SEVERE | Total |  |  |  |  | ABSENT | MILD | MODERATE | SEVERE | Total |  |
| # of Patients | 0 | Frequency | 17 | 16 | 8 | 2 | 43 |  | # of Patients | 0 | Frequency | 9 | 21 | 10 | 3 | 43 |  |
|  |  | Percentage (%) | 39.5% | 37.2% | 18.6% | 4.7% | 100.0% |  |  |  | Percentage (%) | 20.9% | 48.8% | 23.3% | 7.0% | 100.0% |  |
|  | 1 to 5 | Frequency | 10 | 11 | 9 | 3 | 33 |  |  | 1 to 5 | Frequency | 6 | 10 | 15 | 2 | 33 |  |
|  |  | Percentage (%) | 30.3% | 33.3% | 27.3% | 9.1% | 100.0% |  |  |  | Percentage (%) | 18.2% | 30.3% | 45.5% | 6.1% | 100.0% |  |
|  | 5 to 10 | Frequency | 2 | 3 | 2 | 2 | 9 |  |  | 5 to 10 | Frequency | 0 | 5 | 1 | 3 | 9 |  |
|  |  | Percentage (%) | 22.2% | 33.3% | 22.2% | 22.2% | 100.0% |  |  |  | Percentage (%) | 0.0% | 55.6% | 11.1% | 33.3% | 100.0% |  |
|  | 10 to 20 | Frequency | 1 | 4 | 1 | 0 | 6 |  |  | 10 to 20 | Frequency | 1 | 2 | 2 | 1 | 6 |  |
|  |  | Percentage (%) | 16.7% | 66.7% | 16.7% | 0.0% | 100.0% |  |  |  | Percentage (%) | 16.7% | 33.3% | 33.3% | 16.7% | 100.0% |  |
|  | > 20 | Frequency | 7 | 3 | 2 | 0 | 12 |  |  | > 20 | Frequency | 5 | 5 | 1 | 1 | 12 |  |
|  |  | Percentage (%) | 58.3% | 25.0% | 16.7% | 0.0% | 100.0% |  |  |  | Percentage (%) | 41.7% | 41.7% | 8.3% | 8.3% | 100.0% |  |
| Total |  | Frequency | 37 | 37 | 22 | 7 | 103 |  | Total |  | Frequency | 21 | 43 | 29 | 10 | 103 |  |
|  |  | Percentage (%) | 35.9% | 35.9% | 21.4% | 6.8% | 100.0% |  |  |  | Percentage (%) | 20.4% | 41.7% | 28.2% | 9.7% | 100.0% |  |
| ha | Value | df | Sig. Asymptotic (bilateral) | | |  |  |  |  | Value | df | Sig. Asymptotic (bilateral) | | |  |  |  |
| Pearson Chi-square | 11.092^a^ | 12 | 0.521 |  |  |  |  |  | Pearson Chi-square | 19.505^a^ | 12 | 0.077 |  |  |  |  |  |
| Verisimilitude | 10.818 | 12 | 0.545 |  |  |  |  |  | Verisimilitude | 19.195 | 12 | 0.084 |  |  |  |  |  |
| N cases | 103 |  |  |  |  |  |  |  | N cases | 103 |  |  |  |  |  |  |  |
| a. 14 cells (70.0%) have an expected Frequency lower than 5. The expected minimum frequency is .41. | | | | | | | | | a. 13 cells (65.0%) have an expected Frequency lower than 5. The expected minimum frequency is .58. | | | | | | | |  |
|  |  |  |  |  |  |  |  |  |  |  |  |  |  |  |  |  |  |
| **# Patients - Traumatic stress** | | |  |  |  |  |  |  | **# Patients - Cumpulsive** | | |  |  |  |  |  |  |
|  |  |  | ABSENT | MILD | MODERATE | SEVERE | Total |  |  |  |  | ABSENT | MILD | MODERATE | SEVERE | Total |  |
| # of Patients | 0 | Frequency | 31 | 5 | 4 | 3 | 43 |  | # of Patients | 0 | Frequency | 17 | 19 | 6 | 1 | 43 |  |
|  |  | Percentage (%) | 72.1% | 11.6% | 9.3% | 7.0% | 100.0% |  |  |  | Percentage (%) | 39.5% | 44.2% | 14.0% | 2.3% | 100.0% |  |
|  | 1 to 5 | Frequency | 10 | 16 | 3 | 4 | 33 |  |  | 1 to 5 | Frequency | 6 | 13 | 10 | 4 | 33 |  |
|  |  | Percentage (%) | 30.3% | 48.5% | 9.1% | 12.1% | 100.0% |  |  |  | Percentage (%) | 18.2% | 39.4% | 30.3% | 12.1% | 100.0% |  |
|  | 5 to 10 | Frequency | 4 | 3 | 1 | 1 | 9 |  |  | 5 to 10 | Frequency | 4 | 2 | 1 | 2 | 9 |  |
|  |  | Percentage (%) | 44.4% | 33.3% | 11.1% | 11.1% | 100.0% |  |  |  | Percentage (%) | 44.4% | 22.2% | 11.1% | 22.2% | 100.0% |  |
|  | 10 to 20 | Frequency | 1 | 3 | 2 | 0 | 6 |  |  | 10 to 20 | Frequency | 0 | 2 | 3 | 1 | 6 |  |
|  |  | Percentage (%) | 16.7% | 50.0% | 33.3% | 0.0% | 100.0% |  |  |  | Percentage (%) | 0.0% | 33.3% | 50.0% | 16.7% | 100.0% |  |
|  | > 20 | Frequency | 6 | 5 | 1 | 0 | 12 |  |  | > 20 | Frequency | 7 | 4 | 1 | 0 | 12 |  |
|  |  | Percentage (%) | 50.0% | 41.7% | 8.3% | 0.0% | 100.0% |  |  |  | Percentage (%) | 58.3% | 33.3% | 8.3% | 0.0% | 100.0% |  |
| Total |  | Frequency | 52 | 32 | 11 | 8 | 103 |  | Total |  | Frequency | 34 | 40 | 21 | 8 | 103 |  |
|  |  | Percentage (%) | 50.5% | 31.1% | 10.7% | 7.8% | 100.0% |  |  |  | Percentage (%) | 33.0% | 38.8% | 20.4% | 7.8% | 100.0% |  |
|  | Value | df | Sig. Asymptotic (bilateral) | | |  |  |  |  | Value | df | Sig. Asymptotic (bilateral) | | |  |  |  |
| Pearson Chi-square | 23.126^a^ | 12 | 0.027 |  |  |  |  |  | Pearson Chi-square | 21.211^a^ | 12 | 0.047 |  |  |  |  |  |
| Verisimilitude | 24.853 | 12 | 0.016 |  |  |  |  |  | Verisimilitude | 23.172 | 12 | 0.026 |  |  |  |  |  |
| N cases | 103 |  |  |  |  |  |  |  | N cases | 103 |  |  |  |  |  |  |  |
| a. 15 cells (75.0%) have an expected Frequency lower than 5. The expected minimum frequency is .47. | | | | | | | | | a. 14 cells (70.0%) have an expected Frequency lower than 5. The expected minimum frequency is .47. | | | | | | | |  |
