# Supplementary material for: Prevalence of Stress in Healthcare Professionals during the COVID-19 Pandemic in Northeast Mexico: A Remote, Fast Survey Evaluation, Using an Adapted COVID-19 Stress Scales
Source: Int J Environ Res Public Health. 2020 Oct 19;17(20):7624. doi: 10.3390/ijerph17207624 (PMC7593933; doi:10.3390/ijerph17207624)
Supplement: Supplementary file 1 [file ijerph-17-07624-s001.zip › supp table/supp table 7.docx]

| **FOBAP -CSS** | |  |  |  |  |  |  |  | **FOBAP - Danger + Contamination** | | |  |  |  |  |  |
| --- | --- | --- | --- | --- | --- | --- | --- | --- | --- | --- | --- | --- | --- | --- | --- | --- |
|  |  |  | ABSENT | MILD | MODERATE | SEVERE | Total |  |  |  |  | ABSENT | MILD | MODERATE | SEVERE | Total |
| Degree of worry | 0 | Frequency | 5 | 9 | 0 | 0 | 14 |  | Degree of worry | 0 | Frequency | 3 | 7 | 4 | 0 | 14 |
|  |  | Percentage (%) | 35.7% | 64.3% | 0.0% | 0.0% | 100.0% |  |  |  | Percentage (%) | 21.4% | 50.0% | 28.6% | 0.0% | 100.0% |
|  | 1 | Frequency | 2 | 14 | 4 | 1 | 21 |  |  | 1 | Frequency | 3 | 4 | 11 | 3 | 21 |
|  |  | Percentage (%) | 9.5% | 66.7% | 19.0% | 4.8% | 100.0% |  |  |  | Percentage (%) | 14.3% | 19.0% | 52.4% | 14.3% | 100.0% |
|  | 2 | Frequency | 2 | 25 | 5 | 0 | 32 |  |  | 2 | Frequency | 1 | 11 | 18 | 2 | 32 |
|  |  | Percentage (%) | 6.3% | 78.1% | 15.6% | 0.0% | 100.0% |  |  |  | Percentage (%) | 3.1% | 34.4% | 56.3% | 6.3% | 100.0% |
|  | 3 | Frequency | 0 | 11 | 13 | 2 | 26 |  |  | 3 | Frequency | 0 | 4 | 17 | 5 | 26 |
|  |  | Percentage (%) | 0.0% | 42.3% | 50.0% | 7.7% | 100.0% |  |  |  | Percentage (%) | 0.0% | 15.4% | 65.4% | 19.2% | 100.0% |
|  | 4 | Frequency | 0 | 1 | 5 | 2 | 8 |  |  | 4 | Frequency | 0 | 0 | 4 | 4 | 8 |
|  |  | Percentage (%) | 0.0% | 12.5% | 62.5% | 25.0% | 100.0% |  |  |  | Percentage (%) | 0.0% | 0.0% | 50.0% | 50.0% | 100.0% |
| Total |  | Frequency | 9 | 60 | 27 | 5 | 101 |  | Total |  | Frequency | 7 | 26 | 54 | 14 | 101 |
|  |  | Percentage (%) | 8.9% | 59.4% | 26.7% | 5.0% | 100.0% |  |  |  | Percentage (%) | 6.9% | 25.7% | 53.5% | 13.9% | 100.0% |
|  | Value | df | Sig. Asymptotic (bilateral) | | |  |  |  |  | Value | df | Sig. Asymptotic (bilateral) | | |  |  |
| Pearson Chi-square | 44.899^a^ | 12 | 0.000 |  |  |  |  |  | Pearson Chi-square | 30.287^a^ | 12 | 0.003 |  |  |  |  |
| Verisimilitude | 45.344 | 12 | 0.000 |  |  |  |  |  | Verisimilitude | 31.851 | 12 | 0.001 |  |  |  |  |
| linear association | 27.615 | 1 | 0.000 |  |  |  |  |  | linear association | 19.129 | 1 | 0.000 |  |  |  |  |
| N cases | 101 |  |  |  |  |  |  |  | N cases | 101 |  |  |  |  |  |  |
| a. 13 cells (65.0%) have an expected Frequency lower than 5. The expected minimum frequency is .40. | | | | | | | |  | a. 13 cells (65.0%) have an expected Frequency lower than 5. The expected minimum frequency is .55. | | | | | | | |
|  |  |  |  |  |  |  |  |  |  |  |  |  |  |  |  |  |
| **FOBAP - Socioeconomical** | | |  |  |  |  |  |  | **FOBAP - Xenophobia** | |  |  |  |  |  |  |
|  |  |  | ABSENT | MILD | MODERETE | SEVERE | Total |  |  |  |  | ABSENT | MILD | MODERETE | SEVERE | Total |
| Degree of worry | 0 | Frequency | 7 | 4 | 2 | 1 | 14 |  | Degree of worry | 0 | Frequency | 4 | 8 | 2 | 0 | 14 |
|  |  | Percentage (%) | 50.0% | 28.6% | 14.3% | 7.1% | 100.0% |  |  |  | Percentage (%) | 28.6% | 57.1% | 14.3% | 0.0% | 100.0% |
|  | 1 | Frequency | 11 | 5 | 4 | 1 | 21 |  |  | 1 | Frequency | 6 | 8 | 6 | 1 | 21 |
|  |  | Percentage (%) | 52.4% | 23.8% | 19.0% | 4.8% | 100.0% |  |  |  | Percentage (%) | 28.6% | 38.1% | 28.6% | 4.8% | 100.0% |
|  | 2 | Frequency | 12 | 13 | 6 | 1 | 32 |  |  | 2 | Frequency | 6 | 16 | 10 | 0 | 32 |
|  |  | Percentage (%) | 37.5% | 40.6% | 18.8% | 3.1% | 100.0% |  |  |  | Percentage (%) | 18.8% | 50.0% | 31.3% | 0.0% | 100.0% |
|  | 3 | Frequency | 6 | 11 | 6 | 3 | 26 |  |  | 3 | Frequency | 5 | 9 | 7 | 5 | 26 |
|  |  | Percentage (%) | 23.1% | 42.3% | 23.1% | 11.5% | 100.0% |  |  |  | Percentage (%) | 19.2% | 34.6% | 26.9% | 19.2% | 100.0% |
|  | 4 | Frequency | 1 | 3 | 3 | 1 | 8 |  |  | 4 | Frequency | 0 | 2 | 3 | 3 | 8 |
|  |  | Percentage (%) | 12.5% | 37.5% | 37.5% | 12.5% | 100.0% |  |  |  | Percentage (%) | 0.0% | 25.0% | 37.5% | 37.5% | 100.0% |
| Total |  | Frequency | 37 | 36 | 21 | 7 | 101 |  | Total |  | Frequency | 21 | 43 | 28 | 9 | 101 |
|  |  | Percentage (%) | 36.6% | 35.6% | 20.8% | 6.9% | 100.0% |  |  |  | Percentage (%) | 20.8% | 42.6% | 27.7% | 8.9% | 100.0% |
|  | Value | df | Sig. Asymptotic (bilateral) | | |  |  |  |  | Value | df | Sig. Asymptotic (bilateral) | | |  |  |
| Pearson Chi-square | 9.749^a^ | 12 | 0.638 |  |  |  |  |  | Pearson Chi-square | 21.248^a^ | 12 | 0.047 |  |  |  |  |
| Verisimilitude | 10.060 | 12 | 0.611 |  |  |  |  |  | Verisimilitude | 23.125 | 12 | 0.027 |  |  |  |  |
| linear association | 6.310 | 1 | 0.012 |  |  |  |  |  | linear association | 8.568 | 1 | 0.003 |  |  |  |  |
| N cases | 101 |  |  |  |  |  |  |  | N cases | 101 |  |  |  |  |  |  |
| a. 11 cells (55.0%) have an expected Frequency lower than 5. The expected minimum frequency is .55. | | | | | | | |  | a. 11 cells (55.0%) have an expected Frequency lower than 5. The expected minimum frequency is .71. | | | | | | | |
|  |  |  |  |  |  |  |  |  |  |  |  |  |  |  |  |  |
| **FOBAP - Traumatic stress** | |  |  |  |  |  |  |  | **FOBAP - Compulsive** | |  |  |  |  |  |  |
|  |  |  | ABSENT | MILD | MODERETE | SEVERE | Total |  |  |  |  | ABSENT | MILD | MODERETE | SEVERE | Total |
| Degree of worry | 0 | Frequency | 13 | 1 | 0 | 0 | 14 |  | Degree of worry | 0 | Frequency | 6 | 6 | 2 | 0 | 14 |
|  |  | Percentage (%) | 92.9% | 7.1% | 0.0% | 0.0% | 100.0% |  |  |  | Percentage (%) | 42.9% | 42.9% | 14.3% | 0.0% | 100.0% |
|  | 1 | Frequency | 16 | 3 | 0 | 2 | 21 |  |  | 1 | Frequency | 9 | 8 | 3 | 1 | 21 |
|  |  | Percentage (%) | 76.2% | 14.3% | 0.0% | 9.5% | 100.0% |  |  |  | Percentage (%) | 42.9% | 38.1% | 14.3% | 4.8% | 100.0% |
|  | 2 | Frequency | 15 | 11 | 4 | 2 | 32 |  |  | 2 | Frequency | 10 | 17 | 4 | 1 | 32 |
|  |  | Percentage (%) | 46.9% | 34.4% | 12.5% | 6.3% | 100.0% |  |  |  | Percentage (%) | 31.3% | 53.1% | 12.5% | 3.1% | 100.0% |
|  | 3 | Frequency | 6 | 13 | 5 | 2 | 26 |  |  | 3 | Frequency | 7 | 6 | 10 | 3 | 26 |
|  |  | Percentage (%) | 23.1% | 50.0% | 19.2% | 7.7% | 100.0% |  |  |  | Percentage (%) | 26.9% | 23.1% | 38.5% | 11.5% | 100.0% |
|  | 4 | Frequency | 2 | 2 | 2 | 2 | 8 |  |  | 4 | Frequency | 1 | 2 | 2 | 3 | 8 |
|  |  | Percentage (%) | 25.0% | 25.0% | 25.0% | 25.0% | 100.0% |  |  |  | Percentage (%) | 12.5% | 25.0% | 25.0% | 37.5% | 100.0% |
| Total |  | Frequency | 52 | 30 | 11 | 8 | 101 |  | Total |  | Frequency | 33 | 39 | 21 | 8 | 101 |
|  |  | Percentage (%) | 51.5% | 29.7% | 10.9% | 7.9% | 100.0% |  |  |  | Percentage (%) | 32.7% | 38.6% | 20.8% | 7.9% | 100.0% |
|  | Value | df | Sig. Asymptotic (bilateral) | | |  |  |  |  | Value | df | Sig. Asymptotic (bilateral) | | |  |  |
| Pearson Chi-square | 31.670^a^ | 12 | 0.002 |  |  |  |  |  | Pearson Chi-square | 23.524^a^ | 12 | 0.024 |  |  |  |  |
| Verisimilitude | 35.747 | 12 | 0.000 |  |  |  |  |  | Verisimilitude | 20.506 | 12 | 0.058 |  |  |  |  |
| linear association | 22.132 | 1 | 0.000 |  |  |  |  |  | linear association | 8.517 | 1 | 0.004 |  |  |  |  |
| N cases | 101 |  |  |  |  |  |  |  | N cases | 101 |  |  |  |  |  |  |
| a. 13 cells (65.0%) have an expected Frequency lower than 5. The expected minimum frequency is .63. | | | | | | | |  | a. 11 cells (55.0%) have an expected Frequency lower than 5. The expected minimum frequency is .63. | | | | | | | |
