# Supplementary material for: Prevalence of Stress in Healthcare Professionals during the COVID-19 Pandemic in Northeast Mexico: A Remote, Fast Survey Evaluation, Using an Adapted COVID-19 Stress Scales
Source: Int J Environ Res Public Health. 2020 Oct 19;17(20):7624. doi: 10.3390/ijerph17207624 (PMC7593933; doi:10.3390/ijerph17207624)
Supplement: Supplementary file 1 [file ijerph-17-07624-s001.zip › supp table/supp table 8.docx]

| scale construction scoring [#categories(X)-1]/#results(Y) =1 | | | | | |  |  |  |
| --- | --- | --- | --- | --- | --- | --- | --- | --- |
| total of questions per section = 6 | | |  |  |  |  |  |  |
| score/6= category classification score | | | | scoring interval width 1 | | |  |  |
| total of possible points per section (6) score 0 to 4. min score 0, max score 24 | | |  |  |  |  |  |  |
|  |  |  |  |  |  |  |  |  |
| scoring | /#questions | category |  | category | score |  |  |  |
| 0 | 0 | absent |  | absent | 0-6 |  |  |  |
| 1 | 0.166 | absent |  | mild | 7 to 12 |  |  |  |
| 2 | 0.333 | absent |  | moderate | 13 to 18 |  |  |  |
| 3 | 0.5 | absent |  | severe | 19 to 24 |  |  |  |
| 4 | 0.666 | absent |  |  |  |  |  |  |
| 5 | 0.833 | absent |  | category (X) | | | |  |
| 6 | 1 | absent |  | absent | mild | moderate | severe |  |
| 7 | 1.166 | mild |  |  |  |  |  |  |
| 8 | 1.333 | mild |  | scale (results, Y) | | | | |
| 9 | 1.5 | mild |  | 0 | 1 | 2 | 3 | 4 |
| 10 | 1.666 | mild |  |  |  |  |  |  |
| 11 | 1.83 | mild |  |  |  |  |  |  |
| 12 | 2 | mild |  |  |  |  |  |  |
| 13 | 2.166 | moderate |  |  |  |  |  |  |
| 14 | 2.333 | moderate |  |  |  |  |  |  |
| 15 | 2.5 | moderate |  |  |  |  |  |  |
| 16 | 2.666 | moderate |  |  |  |  |  |  |
| 17 | 2.833 | moderate |  |  |  |  |  |  |
| 18 | 3 | moderate |  |  |  |  |  |  |
| 19 | 3.166 | severe |  |  |  |  |  |  |
| 20 | 3.333 | severe |  |  |  |  |  |  |
| 21 | 3.5 | severe |  |  |  |  |  |  |
| 22 | 3.666 | severe |  |  |  |  |  |  |
| 23 | 3.833 | severe |  |  |  |  |  |  |
| 24 | 4 | severe |  |  |  |  |  |  |
